# Evaluation of the causal effects between subjective wellbeing and cardiometabolic health: mendelian randomisation study

**DOI:** 10.1136/bmj.k3788

**Published:** 2018-09-25

**Authors:** Rebecca B Lawn, Louise A C Millard, Neil M Davies, Amy E Taylor, Marcus R Munafò, Nicholas J Timpson, Oliver S P Davis, George Davey Smith, Claire M A Haworth, Robyn E Wootton

**Affiliations:** 1 School of Experimental Psychology, University of Bristol, Bristol BS8 1TU, UK; 2 Department of Population Health Sciences, Bristol Medical School, University of Bristol, Bristol, UK; 3 MRC Integrative Epidemiology Unit, University of Bristol, Bristol, UK; 4 NIHR Biomedical Research Centre at the University Hospitals Bristol NHS Foundation Trust and the University of Bristol, Bristol, UK; 5 UK Centre for Tobacco and Alcohol Studies, University of Bristol, Bristol, UK; 6 Intelligent Systems Laboratory, Department of Computer Science, University of Bristol, Bristol, UK; 7 Avon Longitudinal Study of Parents and Children, Bristol, UK

## Abstract

**Objectives:**

To investigate whether the association between subjective wellbeing (subjective happiness and life satisfaction) and cardiometabolic health is causal.

**Design:**

Two sample, bidirectional mendelian randomisation study.

**Setting:**

Genetic data taken from various cohorts comprised of the general population (mostly individuals of European ancestry, plus a small proportion of other ancestries); follow-up analysis included individuals from the United Kingdom.

**Participants:**

Summary data were used from previous genome wide association studies (number of participants ranging from 83 198 to 339 224), which investigated traits related to cardiovascular or metabolic health, had the largest sample sizes, and consisted of the most similar populations while minimising sample overlap. A follow-up analysis included 337 112 individuals from the UK Biobank (54% female (n=181 363), mean age 56.87 years (standard deviation 8.00) at recruitment).

**Main outcome measures:**

Subjective wellbeing and 11 measures of cardiometabolic health (coronary artery disease; myocardial infarction; total, high density lipoprotein, and low density lipoprotein cholesterol; diastolic and systolic blood pressure; body fat; waist to hip ratio; waist circumference; and body mass index).

**Results:**

Evidence of a causal effect of body mass index on subjective wellbeing was seen; each 1 kg/m^2^ increase in body mass index caused a −0.045 (95% confidence interval −0.084 to −0.006, P=0.02) standard deviation reduction in subjective wellbeing. Follow-up analysis of this association in an independent sample from the UK Biobank provided strong evidence of an effect of body mass index on satisfaction with health (β=−0.035 unit decrease in health satisfaction (95% confidence interval −0.043 to −0.027) per standard deviation increase in body mass index, P<0.001). No clear evidence of a causal effect was seen between subjective wellbeing and the other cardiometabolic health measures, in either direction.

**Conclusions:**

These results suggest that a higher body mass index is associated with a lower subjective wellbeing. A follow-up analysis confirmed this finding, suggesting that the effect in middle aged people could be driven by satisfaction with health. Body mass index is a modifiable determinant, and therefore, this study provides further motivation to tackle the obesity epidemic because of the knock-on effects of higher body mass index on subjective wellbeing.

## Introduction

Subjective wellbeing is most commonly defined as a combination of life satisfaction and happiness (having high positive affect in the absence of negative affect).^1^ Life satisfaction and happiness capture both the cognitive and affective components of subjective wellbeing, respectively. The importance of wellbeing is emphasised by the World Health Organization in their definition of health,^2^ and observational evidence suggests an association between higher subjective wellbeing and better physical health or longevity,^3^ ^4^ ^5^ especially cardiovascular and metabolic health outcomes including cardiovascular disease,^6^ cholesterol levels,^7^ and extremes of body mass index.^8^

More frequently studied, depression has been shown to have the opposite association with cardiometabolic health, increasing the risk of coronary artery disease (especially the chance of a heart attack),^9^ altering serum cholesterol,^10^ and having a U shaped relation with body mass index.^11^ A mendelian randomisation study of body mass index on multiple mental health outcomes found a consistent effect of higher body mass index on increased likelihood of depression, although the effect sizes were small.^12^ This causal effect was replicated in the follow-up analysis of the most recent genome wide association study (GWAS) of depression,^13^ and was replicated using a continuous measure of depressive symptoms.^14^ The study also showed suggestive evidence of a causal link between body mass index and subjective wellbeing using a two sample approach and a relaxed instrument threshold. However, this study did not examine cardiovascular health or other measures of adiposity and did not adjust for sample overlap, so results could be biased towards the confounded observational effect.^15^

Twin analyses suggest partly distinct genetic (and environmental) causes for depression and subjective wellbeing,^16^ which indicates that separate analyses of associations between subjective wellbeing and depression on health outcomes might be appropriate. Observational research suggests that the association between subjective wellbeing and cardiometabolic health goes beyond the absence of negative affect states, reduced arousal, or confounding from socioeconomic position,^17^ and subjective wellbeing is more predictive of health outcomes than negative feelings.^18^ Therefore, subjective wellbeing might be a more effective target for improving cardiometabolic health outcomes than depression. From a public health perspective, it is important to understand whether increasing subjective wellbeing can increase health in later life, given that wellbeing interventions can be cost effective to administer.^19^

**Fig 1:**
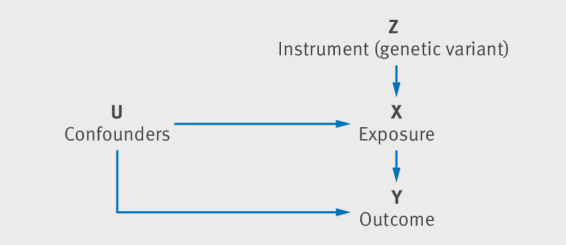
Directed acyclic graph representing the basic instrumental variable analysis in mendelian randomisation

Studies suggesting a link between subjective wellbeing and cardiometabolic health are mostly observational. Owing to reverse causation and residual confounding, causal inferences are difficult to make from observational evidence.^20^ Mendelian randomisation uses genetic variants as instrumental variables for the exposure of interest. The inheritance of specific alleles is largely independent of genetic variants affecting other traits and of conventional disease risk factors, and associations are less prone to other biases inducing reverse causation, because genotype is unchanged over the lifetime.^20^ ^21^ In an instrumental variable analysis, the genetic variant (Z) acts as the instrument that is related to differences in the exposure (X). If the exposure is on the causal pathway with the outcome (Y), then genetic variants that affect the exposure should be associated with the outcome (fig 1).^20^ For example, genetic variants (Z) shown to predispose individuals to have a higher body mass index (exposure X) are associated with lower income, suggesting that increases in body mass index reduce income (outcome Y).^22^ Mendelian randomisation is one method available to triangulate evidence about whether a particular intervention is clinically viable.^23^

The two sample mendelian randomisation method uses summary statistics from each GWAS in one analysis.^24^ Single nucleotide polymorphisms (SNPs) reliably associated with an exposure of interest can essentially be looked up in the GWAS summary statistics of the outcome. For power, multiple genetic variants, rather than single genetic variants, are often used in two sample mendelian randomisation.^15^ Mendelian randomisation requires the genetic instrument to act on the outcome only via the exposure. Genetic variants may be pleiotropic (that is, when one genetic variant affects multiple phenotypes). If the SNPs chosen as instruments act on the outcome via a pathway other than through the exposure (horizontal pleiotropy), estimates can be biased.^20^ ^24^ Use of a larger number of SNPs could protect against this bias if the pleiotropic effects balance out.^15^ Consistent estimates across multiple methods with different assumptions about pleiotropy also ensures that bias is less likely. Further information on the interpretation and assumptions of mendelian randomisation is presented elsewhere.^23^

In this study, we used mendelian randomisation to investigate the association between subjective wellbeing and the cardiometabolic health traits of body mass index, waist to hip ratio, waist circumference, body fat, coronary artery disease, high density lipoprotein cholesterol, low density lipoprotein cholesterol, total cholesterol, systolic blood pressure, diastolic blood pressure, and myocardial infarction. We conducted two sample bidirectional analyses to establish whether subjective wellbeing affects cardiometabolic health traits, or vice versa. We extended previous research by conducting follow-up analysis in an independent sample to avoid sample overlap, adding novel mendelian randomisation methods and examining the causal relation between subjective wellbeing and various cardiometabolic health measures.

## Methods 

Our mendelian randomisation analysis consisted of two parts: two sample mendelian randomisation using GWAS summary data, and a follow-up analysis exploring results with sample overlap.

### Two sample mendelian randomisation

#### Data sources

Details of the contributing GWAS consortiums are listed in table 1. The studies were selected for investigating traits related to cardiovascular or metabolic health, having the largest sample sizes, and consisting of the most similar populations while minimising sample overlap. Percentage sample overlap is presented in supplementary table S1. Subjective wellbeing was measured using any items relating to happiness or positive affect and overall life satisfaction. GWAS of each component were meta-analysed to capture subjective wellbeing.^25^ For further information on the phenotype definitions and GWAS methods for all traits, see supplementary table S2. All phenotype scores were z scored apart from blood pressure.^31^

**Table 1:** Description of GWAS consortiums used for each phenotype

Variable	First author (year)	Consortium	Sample size	Population*	Sex*
Subjective wellbeing	Okbay^25^ (2016)	SSGAC	298 420	100% European	Mixed†
Body mass index	Locke^26^ (2015)	GIANT	339 224	95% European	53% female
Waist to hip ratio	Shungin^27^ (2015)	GIANT	210 088	100% European	56% female
Waist circumference	Shungin^27^ (2015)	GIANT	232 101	100% European	55% female
Body fat percentage	Lu^28^ (2016)	Not available	100 716	89% European	48% female
HDL cholesterol	Willer^29^ (2013)	GLGC	92 860	100% European	Mixed†
LDL cholesterol	Willer^29^ (2013)	GLGC	83 198	100% European	Mixed†
Total cholesterol	Willer^29^ (2013)	GLGC	92 260	100% European	Mixed†
Coronary artery disease	Nikpay^30^ (2015)	CARDIoGRAMplusC4D	Cases=60 801; controls=123 504	77% European	Mixed†
Myocardial infarction	Nikpay^30^ (2015)	CARDIoGRAMplusC4D	Cases=43 676; controls=128 199	Mixed†	Mixed†
Diastolic blood pressure	Wain^31^ (2017)	Not available	150 134	100% European	60% female
Systolic blood pressure	Wain^31^ (2017)	Not available	150 134	100% European	60% female

SSGAC=Social Science Genetics Association Consortium; GLGC=Global Lipids Genetics Consortium; GIANT=Genetic Investigation of Anthropometric Traits consortium; HDL=high density lipoprotein; LDL=low density lipoprotein.

* If not reported, percentage sex and European ancestry were calculated from contributing cohort data in the supplementary materials. All GWAS had similar sex ratios and ancestries included. The largest difference was between the consortiums for coronary artery disease and subjective wellbeing, which used 77% and 100% individuals of European ancestry, respectively. If two populations differ, two sample mendelian randomisation can still be used to test for a causal effect, but the magnitude of the effect might not be as precise.^32^

† Information on the sex ratios and ancestry proportions for the whole sample were not reported or not possible to calculate in the CARDIoGRAMplusC4D, GLGC, and SSGAC consortiums.

#### Statistical analyses

We applied four complementary methods of two sample mendelian randomisation (inverse variance weighted method, mendelian randomisation-Egger (MR-Egger) method, weighted median method, and weighted mode based estimation), which make different assumptions about horizontal pleiotropy. A consistent effect across the four methods is less likely to be a false positive.^33^ If the genetic variants have horizontally pleiotropic effects but are independent of the effects of the genetic variants on the exposure, this is known as balanced pleiotropy. If all the pleiotropic effects are biasing the estimate in the same direction (directional pleiotropy), this will bias the results (with the exception of the MR-Egger method). We used the MR-Egger intercept and MR-PRESSO (mendelian randomisation pleiotropy residual sum and outlier) to test for the presence of directional pleiotropy. Analyses were conducted using MR-Base,^34^ a package for two sample mendelian randomisation.

*Instrument identification*—For all phenotypes, apart from subjective wellbeing, our genetic instruments were composed of genome wide significant SNPs (P<5×10^−8^) from published GWAS studies. Only three genome wide significant SNPs were available for subjective wellbeing. We tested the strength of these instruments by checking whether they predicted happiness in a large independent sample (n=242 219) from the UK Biobank. There was evidence that only one SNP (rs2075677) was associated with happiness (supplementary table S3). Therefore, we used a more liberal P value threshold of P<5×10^−5^ as the instrument for subjective wellbeing. We selected independent SNPs (linkage disequilibrium (LD) R^2^=0.001, >10 000 kb) using the “clump_data” function on MR-Base. If an SNP was unavailable in the outcome GWAS summary statistics, then proxy SNPs were searched for with a minimum LD R^2^=0.8. If a SNP was palindromic, we aligned strands using minor allele frequency up to 0.3, after which 84 SNPs remained associated at P<5×10^−5^. This genetic instrument was checked for overlap with depression^13^ to ensure that the significant SNPs were unique to the subjective wellbeing GWAS. None of the 84 SNPs was associated with major depressive disorder at the genome wide level of significance, suggesting that, based on the current GWAS data, the instruments were independent (supplementary table S4). Power calculations are presented in supplementary table S5.

*Inverse variance weighted method*—We obtained the inverse variance weighted estimate by meta-analysing the SNP specific Wald estimates using multiplicative random effects. The random effects model was chosen to account for heterogeneity, also measured by Cochran’s Q. The Wald estimate is the SNP outcome regression divided by the SNP exposure regression. This method assumes balanced pleiotropy.^35^ ^36^

*MR-Egger method*—This method relaxes the assumptions of mendelian randomisation and allows for directional pleiotropic effects, such that some SNPs could be acting on the outcome through a pathway other than through the exposure. The intercept is not constrained to pass through zero and provides an estimate of the directional pleiotropic effect.^36^ MR-Egger has the lowest power of the four methods to detect a causal effect. It requires variation in the SNP effects, and therefore is most effective when more SNPs are used to create the instrument. MR-Egger is based on the INSIDE assumption (instrument strength independent of the direct effects). It requires that the SNPs’ pleiotropic effects on the outcome are independent of the SNPs’ association with the exposure.^36^ The MR-Egger method is also based on the NOME assumption (no measurement error in the SNP exposure effects),^36^ which is evaluated by the regression dilution I^2^_(GX)_ statistic.^37^ If the statistic was lower than 0.9, simulation extrapolation corrections were performed^37^ (see supplementary table S7 for further information).

*Weighted median method*—This approach assumes that at least 50% of the total weight of the instrument comes from valid variants. It is more likely to give a valid causal estimate than MR-Egger or the inverse variance weighted method because it is more consistent with the true causal effect in the presence of up to 50% invalid variants.^32^

*Weighted mode based estimation*—This method assumes that the most common causal effect is consistent with the true causal effect.^38^ Hence, the remaining instruments could be invalid (that is, violate the assumptions of mendelian randomisation) without biasing the estimated causal effect.

*MR-PRESSO*—This test identifies possible bias from horizontal pleiotropy.^39^ The test consists of three parts: the MR-PRESSO global test, which detects horizontal pleiotropy; the outlier corrected causal estimate, which corrects for the detected horizontal pleiotropy; and the MR-PRESSO distortion test, which estimates whether the causal estimate is significantly different (at P<0.05) after adjustment for outliers.^39^ We conducted all three stages and present the outlier adjusted causal estimates when both global and distortion tests were significant.

### Follow-up analysis in the UK Biobank

We followed up our results from the two sample mendelian randomisation (to overcome potential bias from sample overlap) using a mendelian randomisation analysis where participants for the exposure and outcome were from the same sample (UK Biobank), with a weighted genetic score calculated by use of estimates from GWAS data. The follow-up sample and measures are described below.

#### Study sample

UK Biobank is a national health resource in the United Kingdom with biological measures collected over 10 years (www.ukbiobank.ac.uk). A total of 502 647 participants aged 39-72 years were recruited from across the UK between 2006 and 2010.^40^ After restricting to European ancestry and excluding related individuals, withdrawn consent, and sex mismatched individuals, the study included 337 112 participants.^41^ Of these individuals, the mean age was 56.87 years (standard deviation 8.00) at recruitment, and 54% (n=181 363) were female.

A subsample of participants (n=150 000) were genotyped first, and this sample was selected on the basis of smoking status, to include more current smokers than would be representative of the UK population.^42^ These 150 000 genotyped individuals also contributed measures of happiness to the Social Science Genetics Association Consortium (SSGAC) of subjective wellbeing.^25^ The remaining UK Biobank participants have since been genotyped. To avoid any possible biases associated with smoking, we used the full UK Biobank sample in the follow-up analysis presented here (n=337 112). However, because of partial sample overlap with the SSGAC group, we repeated the same follow-up analysis including only individuals who did not contribute to the consortium (n=242 219).

#### Body mass index allele score and observed body mass index

To further test the association between body mass index and wellbeing, we constructed a polygenic score for body mass index in UK Biobank. This polygenic score was constructed by extracting the 97 variants found to be associated at genome wide significance with body mass index in an independent GWAS^26^ (see supplementary table S6 for full SNP list). Allele scores for each SNP were calculated as a sum of the number of increasing alleles weighted by the effect sizes from the GWAS summary statistics. Therefore, higher polygenic scores indicated an increased risk of higher body mass index. The allele score was standardised to mean zero and standard deviation one. The F statistic was calculated to assess instrument strength in the sample. Observed body mass index was calculated (weight (kg) divided by height (m) squared) from measurements of height and weight taken during the initial assessment centre visit.

#### Outcomes and confounders

We used phenotypic measurements collected at initial assessment (in 2006-10). The measures included subjective happiness, life satisfaction, and baseline demographics. Subjective happiness was assessed by a single item questionnaire measure. Responses to the question “In general, how happy are you?” were collected on a 6 point Likert scale, ranging from “Extremely unhappy” to “Extremely happy.” Individuals who responded with “Do not know” or “Prefer not to answer” were coded as missing.

Life satisfaction was assessed by five single item measures relating to domains of life satisfaction. Domains were: family and relationships, work or job, health, finances, and friendships (eg, “In general, how satisfied are you with your family relationships?”). Responses were collected on a 6 point Likert scale ranging from “Extremely unhappy” to “Extremely happy.” Individuals who responded with “Do not know” or “Prefer not to answer” (as well as “I am not employed” for the work/job domain) were coded as missing.

Baseline demographic measures were collected at initial assessment, including sex, age, and socioeconomic position. Socioeconomic position was measured by the Townsend deprivation index (Townsend, 1987) on the basis of participants’ location in the UK (calculated from output area) and information from the most recent national census.

#### Statistical analysis

We first calculated observational associations using linear regression. We then did mendelian randomisation through instrumental variable regressions run in R,^43^ using the ivreg command from the AER package. We attempted to replicate the effect of body mass index on subjective wellbeing using the body mass index polygenic score as the instrument. Age, sex, and 10 principal components of population structure were controlled for in all instrumental variable regression analyses.

### Patient and public involvement

The current research was not informed by patient and public involvement because it used secondary data. However, future research following on from our findings should be guided by patient and public opinions.

## Results

### Two sample mendelian randomisation

#### Subjective wellbeing predicting cardiometabolic health outcomes

**Fig 2:**
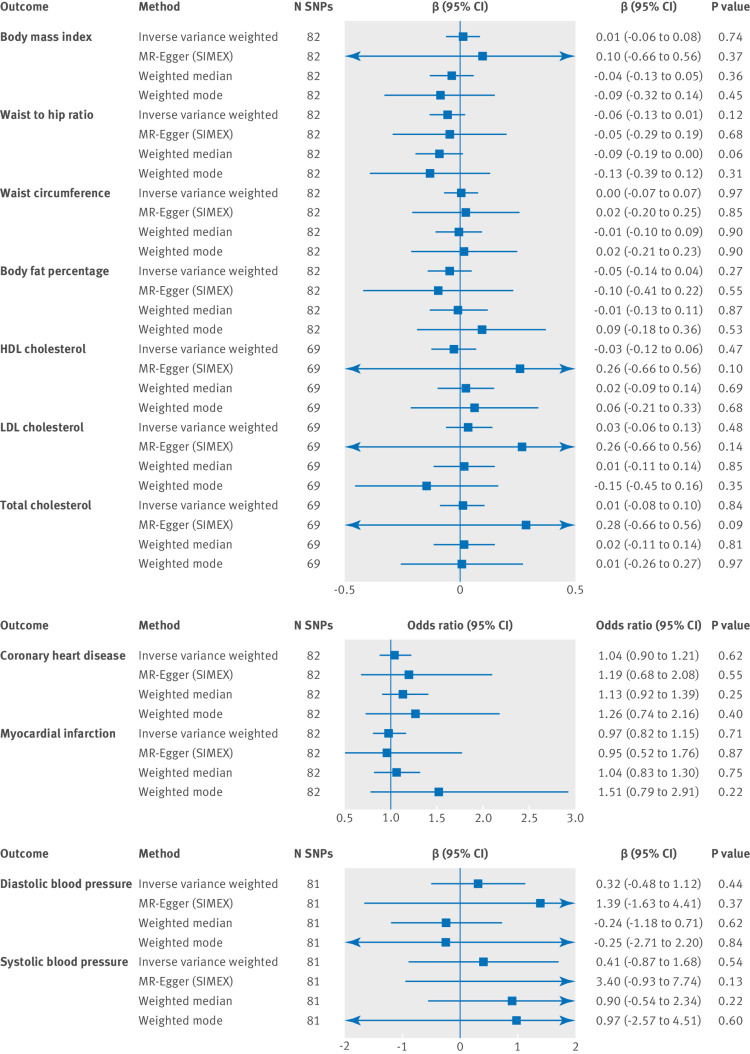
Two sample mendelian randomisation analysis showing the effect of subjective wellbeing on cardiometabolic health outcomes using single nucleotide polymorphisms (SNPs) significant at P<5×10^−5^. One unit increase of subjective wellbeing is equivalent to one standard deviation increase of the subjective wellbeing composite continuous scale. HDL=high density lipoprotein; LDL=low density lipoprotein; N SNP=number of the 84 SNPs associated with wellbeing that were available in the outcome summary statistics (SNPs might be unavailable in the outcome owing to imputation platform or not passing quality control procedures). The regression dilution I^2^_(GX)_ estimate was less than 90% for the subjective wellbeing instrument (see supplementary table S7 for further information); therefore, simulation extrapolation (SIMEX) correction was applied in mendelian randomisation-Egger (MR-Egger) analysis^37^ β values are provided for continuous outcomes and odds ratios are provided for binary outcomes

There was no clear evidence to suggest a causal effect of subjective wellbeing on any of the cardiometabolic health outcomes (fig 2). A more stringent analysis using only genome wide significant SNPs as the instrument produced a similar pattern of results (supplementary figure S1). Phenotype scores were standardised for all outcomes except blood pressure, which is represented on a different scale.

#### Cardiometabolic health predicting subjective wellbeing

**Fig 3:**
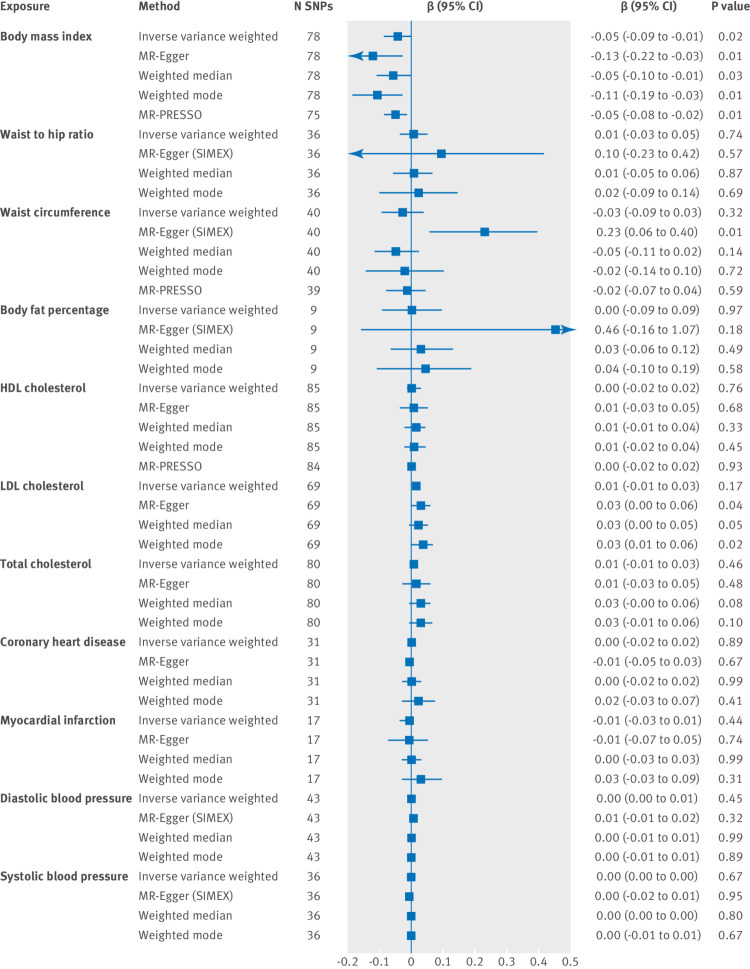
Two sample mendelian randomisation analysis showing the effect of cardiometabolic health exposures on subjective wellbeing per unit of exposure. HDL=high density lipoprotein; LDL=low density lipoprotein; MR-Egger=mendelian randomisation-Egger method; MR-PRESSO=mendelian randomisation pleiotropy residual sum and outlier; N SNPs=number of single nucleotide polymorphisms (SNPs might be unavailable in the outcome owing to imputation platform or not passing quality control procedures). If the regression dilution I^2^_(GX)_ estimates were less than 90%, simulation extrapolation (SIMEX) corrections were applied (supplementary table S7 and supplementary note for further information)

We found evidence that higher body mass index caused lower subjective wellbeing (fig 3). The direction of effect remained consistent across all four methods. There was no clear evidence of a causal effect of any of the cardiovascular health or adiposity exposures on subjective wellbeing (fig 3). Genome wide significant SNPs (P<5×10^−8^) for each cardiometabolic health measure were used as genetic instruments, and the number of SNPs given for each analysis is shown in figure 3.

Cochran’s Q and I^2^ statistics were calculated to check for the presence of heterogeneity (dispersion of SNP effects), which can indicate pleiotropy. We found little evidence of heterogeneity for the association between body mass index and wellbeing (see supplementary table S8 for results and further information). The MR-Egger intercept suggested little evidence of directional pleiotropy (supplementary table S9, all P>0.07). The funnel plot of individual SNP effects showed a symmetrical distribution of SNP effects around the effect estimate, suggesting balanced pleiotropy (supplementary figure S2). We also conducted a leave-one-out analysis, which showed that the SNP with the largest contribution to the effect is rs1421085 located on chromosome 16 in the second intron of the *FTO* (fat mass and obesity associated) gene (supplementary figure S3).

### Follow-up analysis in the UK Biobank

Mean body mass index in the UK Biobank follow-up sample was 27.39 (standard deviation 4.75). Means and standard deviations for the subjective wellbeing measures are given in supplementary table S10, along with observational associations between body mass index and subjective wellbeing in the UK Biobank sample.

#### Association with baseline confounders

The association of the body mass index genetic score and observed body mass index with baseline confounders (age, sex, socioeconomic position, education, smoking, and alcohol consumption) were compared (supplementary figure S4). We found evidence of an association between our body mass index genetic score and socioeconomic position, educational attainment, smoking behaviour, and alcohol consumption. For educational attainment and socioeconomic position, the association was weaker for the genetic score than for the observed body mass index.

#### Association between polygenic score and body mass index

The genetic score was strongly associated with observed body mass index (strength of instrument F(1, 336027)=6237, R^2^=0.018, P<0.001).

#### Follow-up analysis of body mass index (exposure) on subjective wellbeing (outcome)

**Fig 4:**
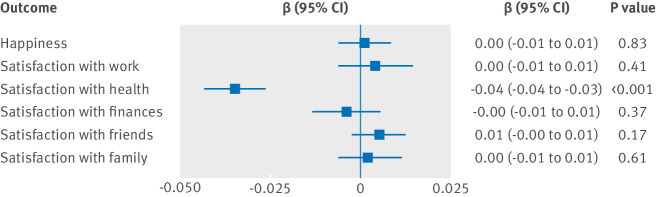
Follow-up analysis, showing causal effects of body mass index on subjective wellbeing

We found strong evidence of a causal effect of body mass index (per 1 kg/m^2^) on satisfaction with health (β=−0.035, 95% confidence interval −0.043 to −0.027, P<0.001; fig 4), which is consistent with previous estimates from an automated hypothesis free MR-PheWAS (which took the polygenic score for body mass index and looked at the association with every outcome in the UK Biobank).^44^ We saw little evidence of a causal effect of body mass index on any of the other measures of subjective wellbeing, and little evidence that this effect differed in older and younger participants (although the age range in UK Biobank is narrow, with all participants over 40 years old). When individuals were split by median age, the evidence for a causal effect of body mass index on satisfaction with health remained strong in both age groups (age ≤58 years, −0.040 (95% confidence interval −0.051 to −0.029), P<0.001; age >58 years, −0.028 (−0.040 to −0.016), P<0.001). When individuals were split by sex, the evidence for a causal effect of body mass index on satisfaction with health remained strong for both sexes (women, −0.034 (−0.044 to −0.024), P<0.001; men, −0.035 (−0.048 to −0.023), P<0.001). The results remained the same in the independent sample after removal of the contributors to the SSGAC consortium (the subjective wellbeing GWAS; supplementary table S11).

## Discussion 

### Evidence for higher body mass index reducing subjective wellbeing

In the present study, we have found evidence indicating that higher body mass index has a causal relation with lower subjective wellbeing, consistent with previous mendelian randomisation findings.^14^ Sensitivity analyses suggested that this association was not due to directional pleiotropy, and the finding was replicated in the UK Biobank cohort. The follow-up analysis suggested that the causal effect of body mass index on subjective wellbeing was driven by satisfaction with health, such that higher body mass index caused lower health satisfaction. 

The pathway from body mass index to health satisfaction could be biological or social. Biological pathways include body mass index as a risk factor for other negative health outcomes such as diabetes, cardiovascular illness, and cancers,^45^ with randomised controlled trials and mendelian randomisation strengthening evidence of a causal effect.^46^ ^47^ ^48^ Therefore, the effect of body mass index on satisfaction with health seen in the current study could reflect accurate perceptions of health. Alternatively, societal influences could cause individuals to associate negative health consequences with a higher body mass index and consequently report lower health satisfaction. Subjective wellbeing and health are in a complex and dynamic system of causal pathways, and further work is needed to understand these pathways using mediation analysis.^3^

Improved subjective wellbeing as an outcome of reducing body mass index might serve as a motivator for behaviour change. Despite knowing the physical health consequences of having a high body mass index, obese individuals often struggle to maintain diets and lose weight.^49^ Individuals value happiness very highly^1^; therefore, emphasis of the potential benefits of weight loss to improve subjective wellbeing could be an unexplored motivator for encouraging weight reduction. Further work should explore the clinical use of subjective wellbeing as a desirable outcome.

In understanding this causal effect further, another important consideration might be the influence of age. Individuals recruited for the UK Biobank were middle aged or older, with an average age of 57 years. Body mass index could be an important determinant of health satisfaction in an older generation as the health implications of obesity (heart disease, diabetes, cancer) begin to emerge.^50^ In younger age groups, body dissatisfaction, self esteem, and bullying might be more important mediators of the association between body mass index and wellbeing.^51^ Further investigation of the causal pathways in a younger sample should be explored, especially as some genetic variants for body mass index show a developmentally specific pattern of association.^52^ ^53^

 Despite causal effects of body mass index seen on subjective wellbeing, there was no causal effect of waist to hip ratio, waist circumference, or body fat percentage. Body mass index is used as a proxy for adiposity because it is widely available, easy to collect in large samples, and other more precise measures of adiposity have not been shown to differ substantially in observational studies.^14^ All confidence intervals for these precise adiposity measures and body mass index overlap; therefore, we cannot rule out a small effect of these phenotypes on subjective wellbeing. Additionally, we might not have seen a clear effect of body fat percentage on subjective wellbeing because the instrument for body fat percentage was relatively weak, using only nine SNPs identified in a GWAS with a relatively small sample size. Furthermore, there are differences in waist to hip ratio and waist circumference between the sexes that we were unable to explore because the GWAS for subjective wellbeing was in a mixed sex sample. Sex differences could have been masking underlying effects of these more precise adiposity measures on subjective wellbeing. Body mass index can also vary for reasons other than adiposity. Future research should therefore explore which aspect of body mass index is driving the identified effect on subjective wellbeing, because a clear understanding of the mechanisms is important for designing interventions.^15^

Observational evidence suggests a non-linear association between mental health and body mass index, where extremely high and low body mass index both predict lower rates of wellbeing.^8^ ^11^ The association between very low body mass index and depression seen in observational studies could be driven by eating disorders such as anorexia nervosa. The two disorders are commonly comorbid, with a 50% lifetime prevalence of major depression in individuals with anorexia.^54^ Twin studies have suggested that this comorbidity is due to shared genetic influence between anorexia and major depressive disorder.^55^ However, our mendelian randomisation estimators assumed a linear relation. Therefore, if individuals with low body mass index also have lower subjective wellbeing, this could lead to the effect observed in mendelian randomisation being smaller than it truly is. New methods to allow for non-linear associations in mendelian randomisation are being developed,^56^ ^57^ but are currently too underpowered to apply here.

### Evidence for subjective wellbeing influencing health outcomes

In our study, the two sample mendelian randomisation analyses showed no clear evidence of a causal effect of subjective wellbeing on cardiometabolic health outcomes. This result is consistent with a prospective analysis in over 700 000 women, which found no effect of happiness on later mortality, if baseline health was controlled for.^58^ Previous observational associations^3^ ^4^ could be due to residual confounding, reverse causation, or publication bias.^59^ ^60^ In our analysis, we saw little evidence that subjective wellbeing had a causal effect on cardiometabolic health outcomes. The genetic variants for subjective wellbeing have small effect sizes, accounting for about 0.01% of the variance, and confidence intervals were wide. Weak instrument bias in mendelian randomisation biases towards the null,^61^ which could explain the lack of evidence for a causal effect of subjective wellbeing. Owing to the direction of bias, false positive effects will not be induced by weak instruments (as could be the case in traditional instrumental variable analysis^61^). Further analysis is needed when stronger instruments are available.

### Evidence for cardiovascular health influencing subjective wellbeing

We saw no clear evidence for a causal effect of cholesterol, coronary artery disease risk, blood pressure, or myocardial infarction risk on subjective wellbeing, meaning that residual confounding is likely to be responsible for the previous observational associations. This conclusion is supported by recent evidence from a new approach called Bayesian direct multimorbidity mapping. This approach found that coronary artery disease was only associated with depression because of an association with body mass index.^62^ However, coronary artery disease and myocardial infarction are rare outcomes, and the SNPs for coronary artery disease risk used in our analysis all had small effect sizes,^30^ resulting in limited power to detect an effect on subjective wellbeing. However, the continuous phenotype of blood pressure still showed no causal effects on subjective wellbeing, further supporting residual confounding. Overall, the evidence suggests no causal effect of cardiovascular health on subjective wellbeing.

### General limitations of the study

In addition to the specific limitations of weak instruments and statistical power outlined above, a more general limitation of this study could be the influence of population structure on the genetic instrument. In large samples such as the UK Biobank cohort, it is difficult to fully remove population structure without removing true effects.^63^ Coincident structure could confound the association between body mass index and subjective wellbeing, generating spurious signal. Although we cannot completely remove the possible influence of structure in our follow-up analysis, we are reasonably confident that the effect of body mass index on satisfaction with health is not spurious because we do not see the same inflation for the negative control outcomes of domain satisfaction or happiness. Furthermore, non-genetic instrumental variables give the same results as genetic instruments in the UK Biobank for educational attainment, a trait largely influenced by structure.^64^

Secondly, the follow-up analysis showed evidence of an association between the body mass index polygenic score and baseline confounders. If the body mass index SNPs affect these confounders independently (horizontal pleiotropy), then the assumptions of mendelian randomisation are violated, and bias would be introduced. Alternatively, alcohol consumption and smoking could be on the causal pathway from body mass index to wellbeing (vertical pleiotropy), which does not violate the assumptions of mendelian randomisation.^65^ ^66^ ^67^ Given the complexity of these genetic and phenotypic relations, it is difficult to disentangle horizontal and vertical pleiotropy; however, we can be reasonably confident that our results are due to vertical pleiotropy, because the MR-Egger intercept did not show evidence of horizontal pleiotropy and MR-PRESSO gave a consistent result after removal of pleiotropic outliers.

### Conclusion

Using mendelian randomisation, we found no clear evidence for a causal effect of cardiovascular health on subjective wellbeing, or of subjective wellbeing on cardiometabolic health. This lack of evidence suggests that previously reported observational associations could have resulted from residual confounding. We found strong evidence for a causal effect of increased body mass index on decreased subjective wellbeing. Follow-up analysis in UK Biobank suggested that the effect of body mass index on subjective wellbeing was driven by an adverse effect of higher body mass index on health satisfaction. Our findings add further support to the need to reduce obesity because of the downstream consequences on mental health and wellbeing. Further work is needed to understand the pathways from body mass index to subjective wellbeing and to explore how the effect of body mass index on mental health varies at different ages.

**What is already known on this topic**

Higher subjective wellbeing is associated with better physical health outcomes, with observational evidence highlighting effects on body mass index and cardiovascular health
Use of genetic data in a mendelian randomisation framework avoids reverse causation and residual confounding (problems associated with observational evidence), hence allowing stronger causal inference
A previous mendelian randomisation analysis has shown evidence of an effect of body mass index on subjective wellbeing, but did not adjust for sample overlap and did not look at other cardiometabolic traits

**What this study adds**

Mendelian randomisation showed strong evidence of a causal effect of higher body mass index on lower subjective wellbeing (β=−0.045 (95% confidence interval −0.084 to −0.006), P=0.02), driven by lower satisfaction with health (β=−0.035 (−0.043 to −0.027), P<0.001)
This study adds further support to the need to prevent obesity because of the downstream consequences on mental health as well as physical health
No clear evidence of a causal association between subjective wellbeing and any other measure of cardiometabolic health was seen in either direction, suggesting that previous associations might be the result of shared confounding by lifestyle factors

## Web extra

Extra material supplied by authors

**Web appendix:** Supplementary materials

## Notes

Contributors: CMAH and OSPD conceived the study. REW conducted the analysis. REW and CMAH drafted the initial manuscript. AET assisted with the two sample mendelian randomisation analysis. LACM assisted with the follow-up analysis, created the polygenic score for body mass index, and provided access to the UK Biobank phenotype data. NMD provided access to the UK Biobank genetic data. RBL assisted with all sensitivity analyses. NMD, GDS, MRM, NJT, LACM, and RBL advised and guided all stages of analysis. All authors assisted with interpretation, commented on drafts of the manuscript, and approved the final version. REW is the guarantor and attests that all listed authors meet authorship criteria and that no others meeting the criteria have been omitted.

Ethical approval: UK Biobank has received ethics approval from the UK National Health Service’s National Research Ethics Service (ref 11/NW/0382).

Data sharing: Scripts are available on GitHub at: https://github.com/MRCIEU/Health-and-Wellbeing-MR.

The lead author affirms that this manuscript is an honest, accurate, and transparent account of the study being reported; that no important aspects of the study have been omitted; and that any discrepancies from the study as planned (and, if relevant, registered) have been explained.

## References

[1] Diener E Subjective well-being. The science of happiness and a proposal for a national index. Am Psychol 2000;55:34-43. 10.1037/0003-066X.55.1.34 11392863

[2] WHO. Mental health: a state of well-being. WHO. 2014. www.who.int/features/factfiles/mental_health/en/ (accessed 30 Jul 2017).

[3] Diener E Pressman SD Hunter J Delgadillo-Chase D If, Why, and When Subjective Well-Being Influences Health, and Future Needed Research. Appl Psychol Health Well Being 2017;9:133-67. 10.1111/aphw.12090 28707767

[4] Steptoe A Dockray S Wardle J Positive affect and psychobiological processes relevant to health. J Pers 2009;77:1747-76. 10.1111/j.1467-6494.2009.00599.x 19796062 PMC2787693

[5] Dockray S Steptoe A Positive affect and psychobiological processes. Neurosci Biobehav Rev 2010;35:69-75. 10.1016/j.neubiorev.2010.01.006 20097225 PMC2895001

[6] Diener E Chan MY Happy people live longer: Subjective well-being contributes to health and longevity. Appl Psychol Health Well-Being 2011;3:1-43 10.1111/j.1758-0854.2010.01045.x .

[7] Ryff CD Singer BH Dienberg Love G Positive health: connecting well-being with biology. Philos Trans R Soc Lond B Biol Sci 2004;359:1383-94. 10.1098/rstb.2004.1521 15347530 PMC1693417

[8] Doll HA Petersen SE Stewart-Brown SL Obesity and physical and emotional well-being: associations between body mass index, chronic illness, and the physical and mental components of the SF-36 questionnaire. Obes Res 2000;8:160-70. 10.1038/oby.2000.17 10757202

[9] Appels A Mental precursors of myocardial infarction. Br J Psychiatry 1990;156:465-71. 10.1192/bjp.156.4.465 1974819

[10] Wardle J Cholesterol and psychological well-being. J Psychosom Res 1995;39:549-62. 10.1016/0022-3999(94)00169-3 7490692

[11] de Wit LM van Straten A van Herten M Penninx BW Cuijpers P Depression and body mass index, a u-shaped association. BMC Public Health 2009;9:14. 10.1186/1471-2458-9-14 19144098 PMC2631467

[12] Hartwig FP Bowden J Loret de Mola C Tovo-Rodrigues L Davey Smith G Horta BL Body mass index and psychiatric disorders: a Mendelian randomization study. Sci Rep 2016;6:32730. 10.1038/srep32730 27601421 PMC5013405

[13] Wray NR Ripke S Mattheisen M eQTLGen 23andMe Major Depressive Disorder Working Group of the Psychiatric Genomics Consortium Genome-wide association analyses identify 44 risk variants and refine the genetic architecture of major depression. Nat Genet 2018;50:668-81. 10.1038/s41588-018-0090-3 29700475 PMC5934326

[14] van den Broek N Treur JL Larsen JK Verhagen M Verweij KJH Vink JM Causal associations between body mass index and mental health: a Mendelian randomisation study. J Epidemiol Community Health 2018;72:708-10. 10.1136/jech-2017-210000 29666151

[15] Burgess S Scott RA Timpson NJ Davey Smith G Thompson SG EPIC- InterAct Consortium Using published data in Mendelian randomization: a blueprint for efficient identification of causal risk factors. Eur J Epidemiol 2015;30:543-52. 10.1007/s10654-015-0011-z 25773750 PMC4516908

[16] Haworth CMA Carter K Eley TC Plomin R Understanding the genetic and environmental specificity and overlap between well-being and internalizing symptoms in adolescence. Dev Sci 2017;20:e12376. 10.1111/desc.12376. 26709037 PMC5347864

[17] Steptoe A Diez Roux AV Happiness, social networks, and health. BMJ 2008;337:a2781. 10.1136/bmj.a2781 19056790

[18] Xu J Roberts RE The power of positive emotions: it’s a matter of life or death--subjective well-being and longevity over 28 years in a general population. Health Psychol 2010;29:9-19. 10.1037/a0016767 20063931

[19] Haworth CM Nelson SK Layous K Stability and change in genetic and environmental influences on well-being in response to an intervention. PLoS One 2016;11:e0155538. 10.1371/journal.pone.0155538 27227410 PMC4881940

[20] Lawlor DA Harbord RM Sterne JA Timpson N Davey Smith G Mendelian randomization: using genes as instruments for making causal inferences in epidemiology. Stat Med 2008;27:1133-63. 10.1002/sim.3034 17886233

[21] Smith GD Ebrahim S ‘Mendelian randomization’: can genetic epidemiology contribute to understanding environmental determinants of disease? Int J Epidemiol 2003;32:1-22. 10.1093/ije/dyg070 12689998

[22] Tyrrell J Jones SE Beaumont R Height, body mass index, and socioeconomic status: mendelian randomisation study in UK Biobank. BMJ 2016;352:i582. 10.1136/bmj.i582 26956984 PMC4783516

[23] Davies NM Holmes MV Davey Smith G Reading Mendelian randomisation studies: a guide, glossary, and checklist for clinicians. BMJ 2018;362:k601. 10.1136/bmj.k601 30002074 PMC6041728

[24] Davey Smith G Hemani G Mendelian randomization: genetic anchors for causal inference in epidemiological studies. Hum Mol Genet 2014;23(R1):R89-98. 10.1093/hmg/ddu328 25064373 PMC4170722

[25] Okbay A Baselmans BM De Neve J-E LifeLines Cohort Study Genetic variants associated with subjective well-being, depressive symptoms, and neuroticism identified through genome-wide analyses [corrections in: *Nat Genet* 2016;48:970 and 2016;48:1591]. Nat Genet 2016;48:624-33. 10.1038/ng.3552 27089181 PMC4884152

[26] Locke AE Kahali B Berndt SI LifeLines Cohort Study ADIPOGen Consortium AGEN-BMI Working Group CARDIOGRAMplusC4D Consortium CKDGen Consortium GLGC ICBP MAGIC Investigators MuTHER Consortium MIGen Consortium PAGE Consortium ReproGen Consortium GENIE Consortium International Endogene Consortium Genetic studies of body mass index yield new insights for obesity biology. Nature 2015;518:197-206. 10.1038/nature14177 25673413 PMC4382211

[27] Shungin D Winkler TW Croteau-Chonka DC ADIPOGen Consortium CARDIOGRAMplusC4D Consortium CKDGen Consortium GEFOS Consortium GENIE Consortium GLGC ICBP International Endogene Consortium LifeLines Cohort Study MAGIC Investigators MuTHER Consortium PAGE Consortium ReproGen Consortium New genetic loci link adipose and insulin biology to body fat distribution. Nature 2015;518:187-96. 10.1038/nature14132 25673412 PMC4338562

[28] Lu Y Day FR Gustafsson S New loci for body fat percentage reveal link between adiposity and cardiometabolic disease risk. Nat Commun 2016;7:10495. 10.1038/ncomms10495 26833246 PMC4740398

[29] Willer CJ Schmidt EM Sengupta S Global Lipids Genetics Consortium Discovery and refinement of loci associated with lipid levels. Nat Genet 2013;45:1274-83. 10.1038/ng.2797 24097068 PMC3838666

[30] Nikpay M Goel A Won HH A comprehensive 1,000 Genomes-based genome-wide association meta-analysis of coronary artery disease. Nat Genet 2015;47:1121-30. 10.1038/ng.3396 26343387 PMC4589895

[31] Wain LV Vaez A Jansen R Novel blood pressure locus and gene discovery using genome-wide association study and expression data sets from blood and the kidney. Hypertension 2017;HYPERTENSIONAHA.117.09438. 28739976 10.1161/HYPERTENSIONAHA.117.09438 PMC5783787

[32] Bowden J Davey Smith G Haycock PC Burgess S Consistent estimation in Mendelian randomization with some invalid instruments using a weighted median estimator. Genet Epidemiol 2016;40:304-14. 10.1002/gepi.21965 27061298 PMC4849733

[33] Lawlor DA Tilling K Davey Smith G Triangulation in aetiological epidemiology. Int J Epidemiol 2016;45:1866-86. 28108528 10.1093/ije/dyw314 PMC5841843

[34] Hemani G Zheng J Elsworth B The MR-Base platform supports systematic causal inference across the human phenome. Elife 2018;7:e34408. 10.7554/eLife.34408 29846171 PMC5976434

[35] Burgess S Butterworth A Thompson SG Mendelian randomization analysis with multiple genetic variants using summarized data. Genet Epidemiol 2013;37:658-65. 10.1002/gepi.21758 24114802 PMC4377079

[36] Bowden J Davey Smith G Burgess S Mendelian randomization with invalid instruments: effect estimation and bias detection through Egger regression. Int J Epidemiol 2015;44:512-25. 10.1093/ije/dyv080 26050253 PMC4469799

[37] Bowden J Del Greco M F Minelli C Davey Smith G Sheehan NA Thompson JR Assessing the suitability of summary data for two-sample Mendelian randomization analyses using MR-Egger regression: the role of the I2 statistic. Int J Epidemiol 2016;45:1961-74. 27616674 10.1093/ije/dyw220 PMC5446088

[38] Hartwig FP Davey Smith G Bowden J Robust inference in summary data Mendelian randomization via the zero modal pleiotropy assumption. Int J Epidemiol 2017;46:1985-98. 10.1093/ije/dyx102 29040600 PMC5837715

[39] Verbanck M Chen C-Y Neale B Do R Detection of widespread horizontal pleiotropy in causal relationships inferred from Mendelian randomization between complex traits and diseases [correction in: *Nat Genet* 2018;50:1196]. Nat Genet 2018;50:693-8. 10.1038/s41588-018-0099-7 29686387 PMC6083837

[40] Sudlow C Gallacher J Allen N UK biobank: an open access resource for identifying the causes of a wide range of complex diseases of middle and old age. PLoS Med 2015;12:e1001779. 10.1371/journal.pmed.1001779 25826379 PMC4380465

[41] Mitchell R, Hemani G, Dudding T, Paternoster L. UK Biobank Genetic Data: MRC-IEU Quality Control, Version 1. https://data.bris.ac.uk/data/dataset/3074krb6t2frj29yh2b03x3wxj 2017.

[42] Wain LV Shrine N Miller S UK Brain Expression Consortium (UKBEC) OxGSK Consortium Novel insights into the genetics of smoking behaviour, lung function, and chronic obstructive pulmonary disease (UK BiLEVE): a genetic association study in UK Biobank. Lancet Respir Med 2015;3:769-81. 10.1016/S2213-2600(15)00283-0 26423011 PMC4593935

[43] R. Core Team. *R: A language and environment for statistical computing. R Foundation for Statistical Computing, Vienna, Austria. 2013*. ISBN 3-900051-07-0 2014.

[44] Millard LAC Davies NM Gaunt TR Davey Smith G Tilling K Software Application Profile: PHESANT: a tool for performing automated phenome scans in UK Biobank. Int J Epidemiol 2017. 29040602 10.1093/ije/dyx204 PMC5837456

[45] World Health Organization Obesity: preventing and managing the global epidemic. World Health Organization, 2000. 11234459

[46] Holmes MV Lange LA Palmer T Causal effects of body mass index on cardiometabolic traits and events: a Mendelian randomization analysis [correction in: *Am J Hum Genet* 2014;94:312]. Am J Hum Genet 2014;94:198-208. 10.1016/j.ajhg.2013.12.014 24462370 PMC3928659

[47] Johnston CA Moreno JP Foreyt JP Cardiovascular effects of intensive lifestyle intervention in type 2 diabetes. Curr Atheroscler Rep 2014;16:457. 10.1007/s11883-014-0457-6 25288176 PMC5321176

[48] Neter JE Stam BE Kok FJ Grobbee DE Geleijnse JM Influence of weight reduction on blood pressure: a meta-analysis of randomized controlled trials. Hypertension 2003;42:878-84. 10.1161/01.HYP.0000094221.86888.AE 12975389

[49] Thomas SL Hyde J Karunaratne A Kausman R Komesaroff PA “They all work...when you stick to them”: a qualitative investigation of dieting, weight loss, and physical exercise, in obese individuals. Nutr J 2008;7:34. 10.1186/1475-2891-7-34 19025661 PMC2607302

[50] Rosamond W Flegal K Friday G American Heart Association Statistics Committee and Stroke Statistics Subcommittee Heart disease and stroke statistics--2007 update: a report from the American Heart Association Statistics Committee and Stroke Statistics Subcommittee [corrections in: *Circulation* 2007;115:e172 and 2010;122:e9]. Circulation 2007;115:e69-171. 10.1161/CIRCULATIONAHA.106.179918 17194875

[51] Sujoldzić A De Lucia A A cross-cultural study of adolescents--BMI, body image and psychological well-being. Coll Antropol 2007;31:123-30. 17598390

[52] Haworth CM Carnell S Meaburn EL Davis OS Plomin R Wardle J Increasing heritability of BMI and stronger associations with the FTO gene over childhood. Obesity (Silver Spring) 2008;16:2663-8. 10.1038/oby.2008.434 18846049

[53] Rosenquist JN Lehrer SF O’Malley AJ Zaslavsky AM Smoller JW Christakis NA Cohort of birth modifies the association between FTO genotype and BMI. Proc Natl Acad Sci U S A 2015;112:354-9. 10.1073/pnas.1411893111 25548176 PMC4299180

[54] Kennedy SH Kaplan AS Garfinkel PE Rockert W Toner B Abbey SE Depression in anorexia nervosa and bulimia nervosa: discriminating depressive symptoms and episodes. J Psychosom Res 1994;38:773-82. 10.1016/0022-3999(94)90030-2 7877132

[55] Wade TD Bulik CM Neale M Kendler KS Anorexia nervosa and major depression: shared genetic and environmental risk factors. Am J Psychiatry 2000;157:469-71. 10.1176/appi.ajp.157.3.469 10698830

[56] Silverwood RJ Holmes MV Dale CE Alcohol-ADH1B Consortium Testing for non-linear causal effects using a binary genotype in a Mendelian randomization study: application to alcohol and cardiovascular traits. Int J Epidemiol 2014;43:1781-90. 10.1093/ije/dyu187 25192829 PMC4276061

[57] Burgess S Davies NM Thompson SG EPIC-InterAct Consortium Instrumental variable analysis with a nonlinear exposure-outcome relationship. Epidemiology 2014;25:877-85. 10.1097/EDE.0000000000000161 25166881 PMC4222800

[58] Liu B Floud S Pirie K Green J Peto R Beral V Million Women Study Collaborators Does happiness itself directly affect mortality? The prospective UK Million Women Study. Lancet 2016;387:874-81. 10.1016/S0140-6736(15)01087-9 26684609 PMC5075047

[59] Ioannidis JP Munafò MR Fusar-Poli P Nosek BA David SP Publication and other reporting biases in cognitive sciences: detection, prevalence, and prevention. Trends Cogn Sci 2014;18:235-41. 10.1016/j.tics.2014.02.010 24656991 PMC4078993

[60] Macleod J Davey Smith G Psychosocial factors and public health: a suitable case for treatment? J Epidemiol Community Health 2003;57:565-70. 10.1136/jech.57.8.565 12883057 PMC1732553

[61] Burgess S Thompson SG CRP CHD Genetics Collaboration Avoiding bias from weak instruments in Mendelian randomization studies. Int J Epidemiol 2011;40:755-64. 10.1093/ije/dyr036 21414999

[62] Marx P Antal P Bolgar B Bagdy G Deakin B Juhasz G Comorbidities in the diseasome are more apparent than real: What Bayesian filtering reveals about the comorbidities of depression. PLoS Comput Biol 2017;13:e1005487. 10.1371/journal.pcbi.1005487 28644851 PMC5507322

[63] Haworth S Mitchell R Corbin L Common genetic variants and health outcomes appear geographically structured in the UK Biobank sample: Old concerns returning and their implications. bioRxiv 2018;294876 10.1101/294876.

[64] Davies NM Dickson M Smith GD The causal effects of education on health outcomes in the UK Biobank. Nat Hum Behav 2018;2:117-25. 10.1038/s41562-017-0279-y . PMC6217998 30406209

[65] Clarke T-K Adams MJ Davies G Genome-wide association study of alcohol consumption and genetic overlap with other health-related traits in UK Biobank (N=112 117). Mol Psychiatry 2017;22:1376-84. 10.1038/mp.2017.153 28937693 PMC5622124

[66] Clarke T-K McIntosh AM Response to’Problems in interpreting and using GWAS of conditional phenotypes illustrated by alcohol GWAS’. bioRxiv 2018;290965 10.1101/290965. PMC6004313 29520038

[67] Taylor A Richmond R Palviainen T The effect of body mass index on smoking behaviour and nicotine metabolism: a Mendelian randomization study. bioRxiv 2018;299834 10.1101/299834. PMC6452214 30561638

