# Supplementary material for: Evaluation of the causal effects between subjective wellbeing and cardiometabolic health: mendelian randomisation study
Source: BMJ. 2018 Sep 25;362:k3788. doi: 10.1136/bmj.k3788 (PMC6155050; doi:10.1136/bmj.k3788)
Supplement: Supplementary file 1 — Web appendix: Supplementary materials [file woor044745.ww.pdf]

Evaluating the causal associations between subjective wellbeing and cardiometabolic health: a Mendelian randomisation study

Authors: Robyn E Wootton, postdoctoral researcher[1,3], Rebecca B Lawn, doctoral researcher[1,3], Louise A C Millard, research fellow[2,3,6], Neil M Davies, research fellow[2,3], Amy Taylor, research fellow[2,4], Marcus R Munafò, professor[1,3,5], Nicholas J Timpson, professor[2,3,7], Oliver S P Davis, reader[2,3], George Davey Smith, professor[2,3], Claire M A Haworth, reader[1, 2, 3].

Author Affiliations:
[1]School of Experimental Psychology, University of Bristol, Bristol, BS8 1TU
[2]Department of Population Health Sciences, Bristol Medical School, University of Bristol, Bristol, BS8 2PR
[3]MRC Integrative Epidemiology Unit, University of Bristol, Bristol, BS8 2PR
[4]NIHR Biomedical Research Centre at the University Hospitals Bristol NHS Foundation Trust and the University of Bristol, (BS8 2BN).
[5]UK Centre for Tobacco and Alcohol Studies, University of Bristol, Bristol, BS8 1TU
[6]Intelligent Systems Laboratory, Department of Computer Science, University of Bristol, Bristol, BS8 1UB
[7]Avon Longitudinal Study of Parents and Children, Oakfield House, Oakfield Grove, Bristol, BS8 2BN

<u>Index of Supplementary Figures</u>

Figure S1. Two-sample MR of subjective wellbeing on cardiometabolic health using genome-wide significant SNPs as the instrument — Page 2
Figure S2. Funnel plot of individual SNP effects of BMI on wellbeing. — Page 4
Figure S3. Leave-one-out analysis — Page 5
Figure S4. Bias plots of association with baseline confounders — Page 6

<u>Index of Supplementary Tables</u>

Table S1. Percentage sample overlap between the SSGAC GWAS for subjective wellbeing and each of the GWAS for cardiometabolic health — Page 8
Table S2. Descriptions of GWAS demographics — Page 9
Table S3. Linear regression of the three genome-wide significant SNPs for subjective wellbeing predicting happiness in the UK Biobank independent sample (N= 242,219) — Page 11
Table S4. Overlap between SNPs for subjective wellbeing and Major Depressive Disorder — Page 12
Table S5. Power calculation for two-sample Mendelian randomisation — Page 16
Table S6. SNPs associated with BMI at the genome-wide level of significance — Page 17
Table S7. Regression dilution I^2 statistics — Page 20
Table S8. Tests of heterogeneity for cardiometabolic health traits as the exposure and subjective wellbeing as the outcome — Page 21
Table S9. Tests of directional pleiotropic effects of the genetic instruments for physical health on subjective wellbeing — Page 22
Table S10. Linear regressions between BMI and subjective wellbeing in the UK Biobank sample — Page 23
Table S11. Results of one-sample MR of BMI (exposure) on subjective wellbeing (outcome) in the independent UK Biobank sample — Page 24

Supplementary Tables and Figures

Figure S1. Two-sample MR analysis: the effect of subjective wellbeing on cardiometabolic health outcomes using 3 genome-wide significant variants as the instrument for subjective wellbeing.

Outcome	Method	Beta (95% CI)	P value
Body mass index	Inverse variance weighted	0.252 (−0.144, 0.648)	0.212
	Weighted median	0.367 (−0.011, 0.745)	0.057
Waist−to−Hip Ratio	Inverse variance weighted	0.007 (−0.320, 0.334)	0.966
	Weighted median	−0.002 (−0.409, 0.404)	0.991
Waist Circumference	Inverse variance weighted	0.070 (−0.348, 0.487)	0.743
	Weighted median	−0.142 (−0.549, 0.264)	0.492
Body Fat	Inverse variance weighted	0.492 (0.091, 0.892)	0.016
	Weighted median	0.384 (−0.138, 0.907)	0.149
HDL cholesterol	Inverse variance weighted	0.134 (−0.366, 0.634)	0.599
	Weighted median	0.229 (−0.253, 0.711)	0.351
LDL cholesterol	Inverse variance weighted	−0.025 (−0.429, 0.379)	0.905
	Weighted median	−0.219 (−0.736, 0.298)	0.406
Total cholesterol	Inverse variance weighted	0.050 (−0.326, 0.426)	0.792
	Weighted median	0.039 (−0.404, 0.482)	0.862

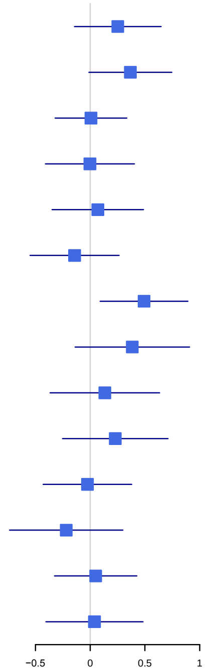

	Method	Beta (95% CI)	P value
Diastolic Blood Pressure	Inverse variance weighted	2.681 (−2.593, 7.955)	0.319
	Weighted median	4.146 (0.203, 8.088)	0.039
Systolic Blood Pressure	Inverse variance weighted	4.183 (−0.501, 8.867)	0.080
	Weighted median	5.142 (−0.482, 10.766)	0.073

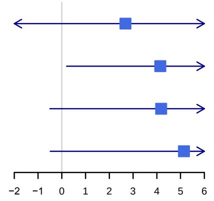

		OR (95% CI)	P value
CAD	Inverse variance weighted	1.421 (0.702, 2.876)	0.329
	Weighted median	1.181 (0.506, 2.756)	0.700
Myocardial infarction	Inverse variance weighted	1.239 (0.567, 2.708)	0.593
	Weighted median	1.228 (0.461, 3.271)	0.682

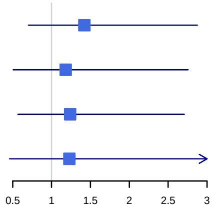

The genetic instrument for the exposure, subjective wellbeing, was 3 genome-wide significant SNPs that each explain 0.1% of the variance, identified by the SSGAC [1]. Due to a restricted number of SNPs, MR-Egger, MBE and MR-PRESSO could not be conducted.

One unit increase of subjective wellbeing is equivalent to one standard deviation increase of the subjective wellbeing composite continuous scale. The genetic instrument was the 3 genome-wide significant SNPs for subjective wellbeing from Okbay et al [1]. Suitable proxies were identified at an LD cut-off $R^2>0.8$. Arrows on confidence intervals indicate they extend beyond the axis. Phenotype scores for all measures were standardised apart from for blood pressure which is represented on a different scale. There was no clear evidence to suggest a causal effect of subjective wellbeing on any of the health outcomes (see Figure 2).

Figure S2. Funnel plot of individual SNP effects of BMI on wellbeing.

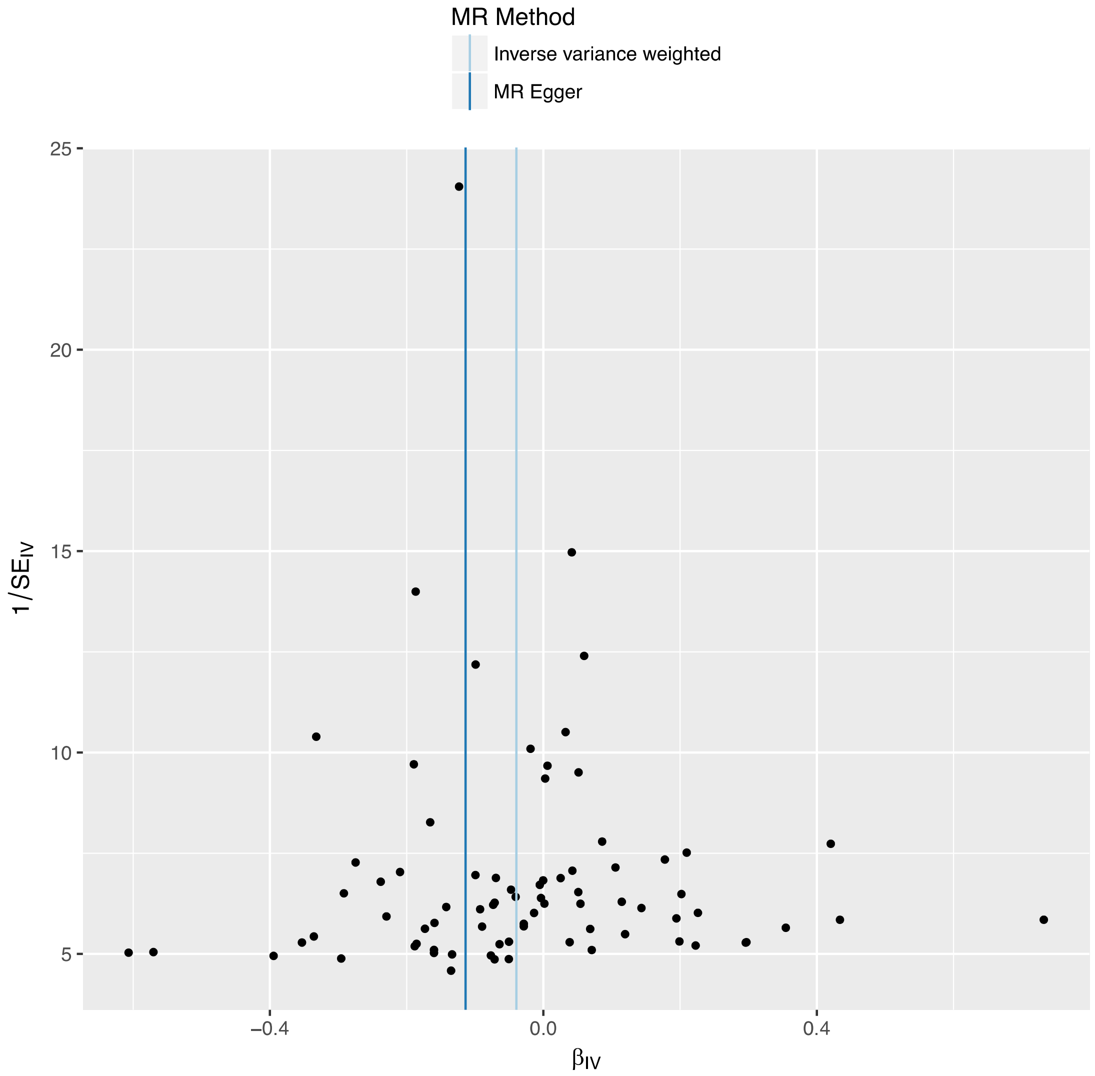

MR Egger and IV weighted estimates are represented with blue lines. On the x-axis, β_{IV} represents the effect size of each SNP. On the y-axis, $1/SE_{IV}$ represents the inverse standard error for each SNP effect.

Figure S3. Leave-one-out analysis: each row represents a two-sample MR analysis of BMI on subjective wellbeing using all of the genome-wide significant SNPs available from Locke et al. [2] except for the SNP listed on the y-axis. The point represents the effect size with that SNP removed and the line represents the standard error.

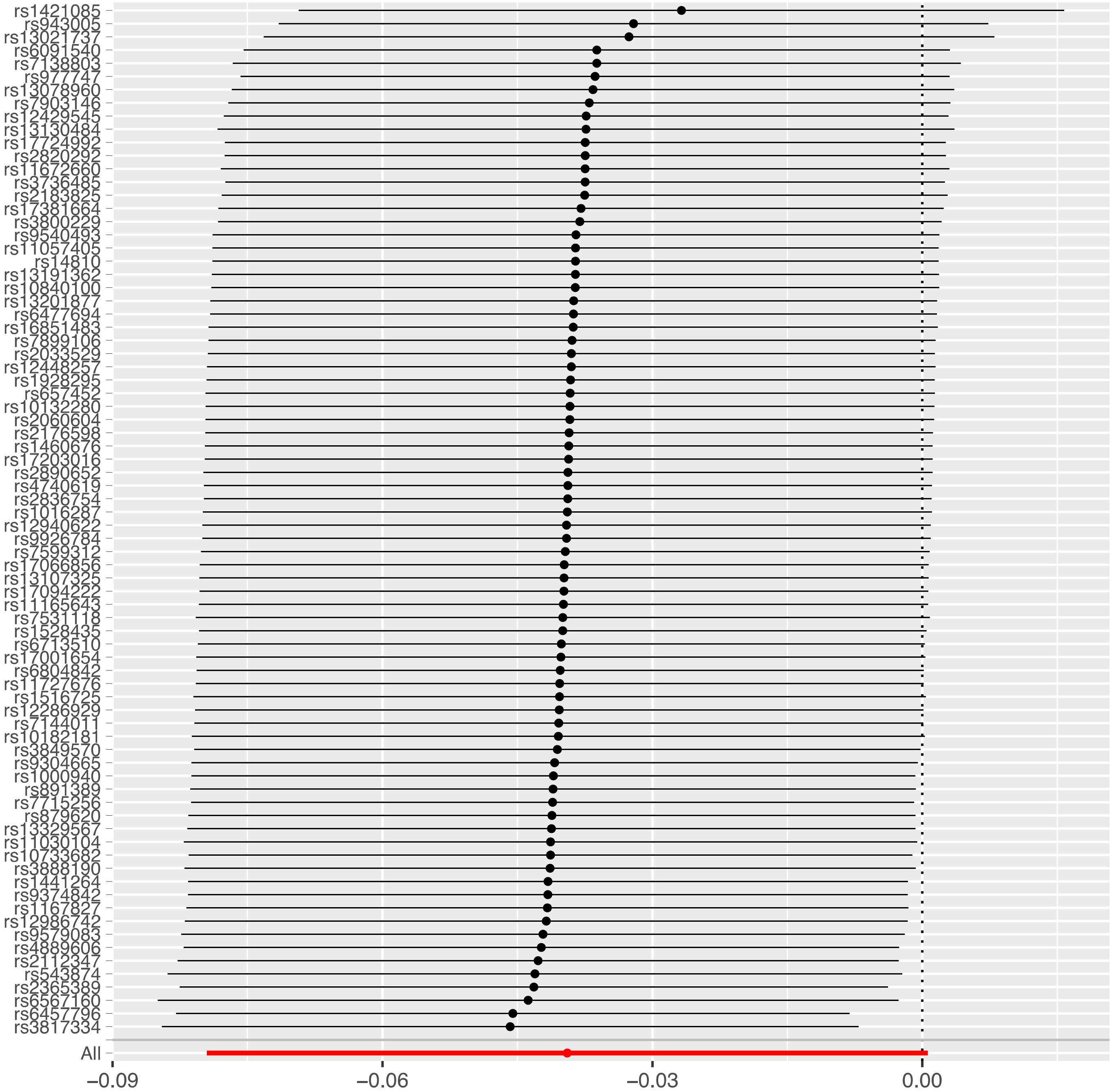

Leave-one-out analysis was conducted using MR Base [3] to identify if any individual SNPs were driving the association between BMI and wellbeing. Results are shown in Figure S2. The SNP with the largest contribution to the effect is rs1421085 located on chromosome 16 in the second intron of the FTO (fat mass and obesity associated) gene. FTO has been repeatedly associated with obesity in different populations [4]. However, the biological consequences of intronic FTO SNPs are still unknown. They are currently thought to play a regulatory role in FTO gene expression in the hypothalamus [5].

Although research is not completely certain of the role of FTO, its large effect size and robust association with obesity suggest that this gene has the largest effect in the two-sample MR because of its BMI effect size rather than because of pleiotropic effects.

Figure S4. Bias plots of association with baseline confounders for BMI, comparing observational and MR analyses

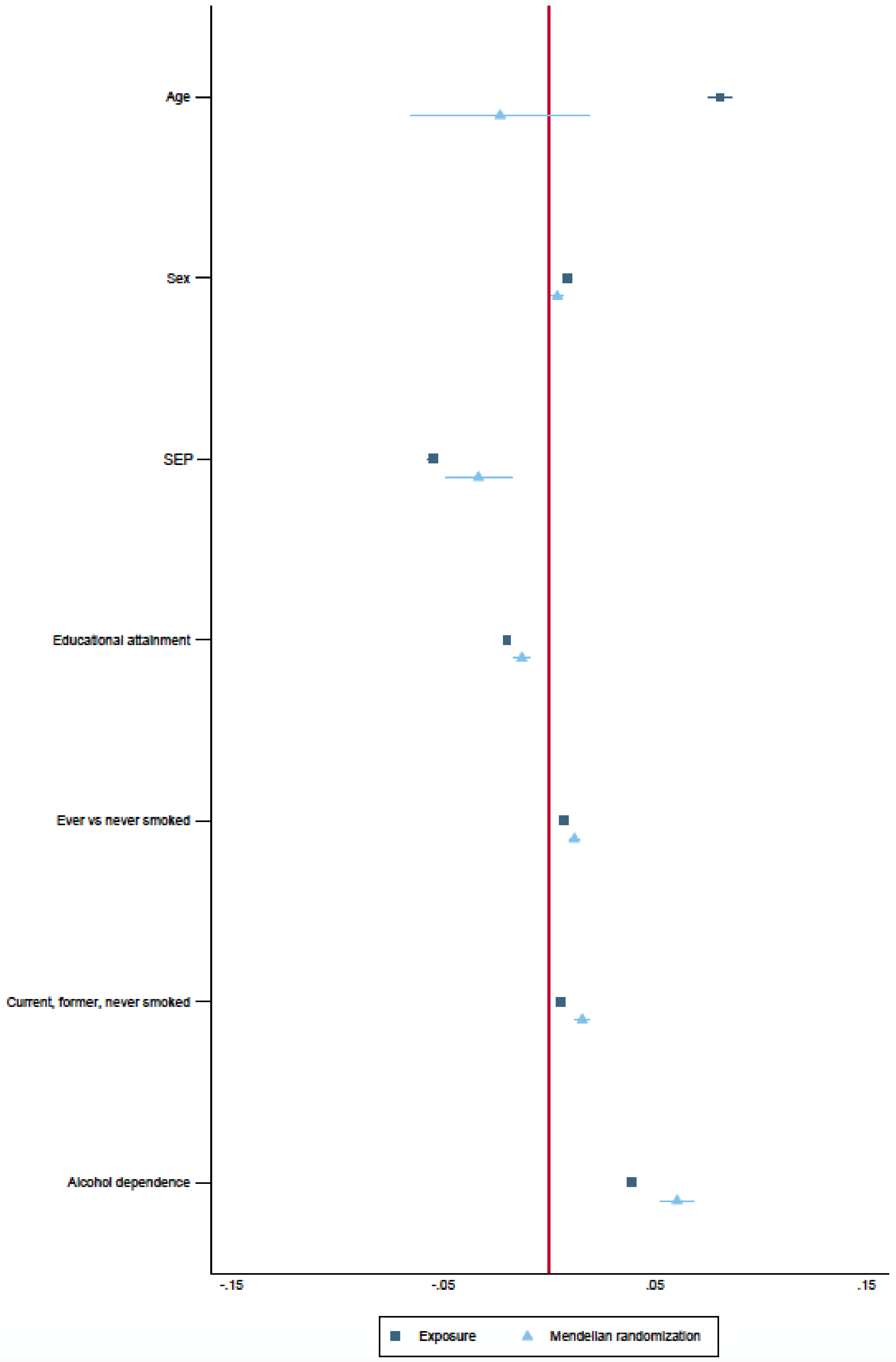

Table S1. Percentage sample overlap between the SSGAC GWAS for subjective wellbeing [1] and each of the GWAS for cardiometabolic health

**Trait**			**GWAS Consortia**	**% Sample Overlap**
**Coronary Artery Disease**	Nikpay [6]	2015	CARDIoGRAMplusC4D	6%
**Total Cholesterol**	Willer [7]	2013	GLGC	4%
**HDL Cholesterol**	Willer [7]	2013	GLGC	4%
**LDL Cholesterol**	Willer [7]	2013	GLGC	4%
**Myocardial Infarction**	Nikpay [6]	2015	CARDIoGRAMplusC4D	6%
**Diastolic Blood Pressure**	Wain [8]	2017	N/A	19%
**Systolic Blood Pressure**	Wain [8]	2017	N/A	19%
**BMI**	Locke [2]	2015	GIANT	36%
**Waist to Hip Ratio**	Shungin [9]	2015	GIANT	32%
**Waist Circumference**	Shungin [9]	2015	GIANT	32%
**Body Fat**	Lu [10]	2016	N/A	9%

Table S2. Descriptions of GWAS demographics

Phenotype	GWAS Phenotype Definition	Mean Age (years)	Covariates	Population Stratification	Instrument F statistic
**Subjective Wellbeing**	Any items of scales capturing positive affect or life satisfaction (e.g. “During the past week, I was happy?” and “How satisfied are you with your life as a whole?” respectively). The two were pooled and equally weighted.	-	sex, age, age^2 and cohort specific covariates e.g. batch effects	At least 4 PCs	20.00
**Coronary Artery Disease**	Criteria for defining cases is given separately for each cohort in the cohort descriptions supplementary note [6]. Of the CAD cases, ~70% had a reported history of MI.	-	Given for each cohort in the cohort descriptions supplementary note [6].	Given for each cohort in the cohort descriptions supplementary note [6].	60.69
**Myocardial Infarction**		-			59.97
**Total Cholesterol**	Where possible, individuals on lipid lowering medication were excluded. The majority of studies measured lipids after >8 hours of fasting. 24% of studies directly measured cholesterol and the rest estimated it using the Friedewald formula. Total cholesterol is calculated from HDL, LDL and triglycerides.	-	Sex, age, age^2	24% of cohorts controlled for population structure using either PC or mixed model approaches	138.38
**HDL Cholesterol**		-			119.75
**LDL Cholesterol**		-			159.76
**Diastolic Blood Pressure**	Diastolic blood pressure measured in mmHg. 10mmHg was added to their score if they were known to be taking antihypertensive medication. Specific conditions differed for each cohort, details can be found in Supplementary materials of [8].	53.13	sex, age, age^2, BMI	Each cohort adjusted as necessary	48.59

**Systolic Blood Pressure**	Systolic blood pressure measured in mmHg. 15mmHg was added to their score if they were known to be taking antihypertensive medication. Specific conditions differed for each cohort, details can be found in Supplementary materials of [8].	53.13	sex, age, age^2, BMI	Each cohort adjusted as necessary	49.22
**BMI**	Combination of measured or self-reported BMI. Weight in kg per height in m^2.	56.39	Sex, age, age^2, BMI and cohort-specific covariates if necessary	Each cohort adjusted as necessary	66.83
**Waist to Hip Ratio**	The ratio of waist circumference to hip circumference adjusted for BMI.	54.39	Sex, age, age^2, BMI and cohort-specific covariates if necessary	Each cohort adjusted as necessary	54.07
**Waist Circumference**	Waist circumference adjusted for BMI.	54.39			59.85
**Body Fat %**	Body fat percentage was measured using either bioimpedance analysis or dual energy X-ray absorptiometry (DEXA).	-	Sex, age, age2 and study-specific covariates	Each cohort adjusted as necessary	44.53

Note. For further description of all phenotypes see individual papers supplementary materials. Mean F statistic of greater than 10% indicates a suitable instrument [11].

Table S3. Linear regression of the three genome-wide significant SNPs for subjective wellbeing predicting happiness in the UK Biobank independent sample (N= 242,219).

	Effect allele	Beta (95% CI)	p-value
**rs2075677**	G	-0.013 (-0.021, -0.005)	0.001
**rs4958581**	C	-0.001 (-0.009, 0.007)	0.814
**rs3756290**	A	-0.002 (-0.010, 0.006)	0.657

Note. The first release of genetic data from the UK Biobank (~150,000) was part of the SSGAC discovery GWAS [1] therefore genetic data from the second release (~350,000) was used in this independent analysis.

Table S4. Overlap between SNPs associated with subjective wellbeing at $p<5x10^{-5}$ and their association with Major Depressive Disorder

						Subjective Wellbeing			Major Depressive Disorder		
**SNP**	**CHR**	**BP**	**A1**	**A2**	**EAF**	**Beta**	**SE**	**P-value**	**OR**	**SE**	**P-value**
**rs10172421**	2	204238402	A	C	0.105	-0.021	0.005	1.11E-05	1.002	0.012	0.867
**rs1028144**	2	99279354	A	G	0.530	0.013	0.003	3.97E-05	0.989	0.008	0.160
**rs1030443**	20	17408386	C	G	0.856	-0.019	0.005	3.33E-05	1.024	0.011	0.035
**rs1075737**	7	103071193	A	G	0.185	-0.019	0.004	8.11E-06	1.024	0.011	0.026
**rs10769190**	11	46151490	T	C	0.198	0.018	0.004	2.19E-06	0.991	0.010	0.361
**rs10895581**	11	104002184	T	C	0.797	0.016	0.004	3.25E-05	0.987	0.010	0.170
**rs10923025**	1	88579746	T	G	0.946	-0.029	0.007	3.05E-05	0.990	0.018	0.573
**rs11043207**	12	122268696	C	G	0.797	0.018	0.004	3.84E-05	0.997	0.011	0.812
**rs11073619**	15	85188839	T	C	0.105	0.025	0.005	3.42E-06	1.024	0.013	0.074
**rs1135436**	10	63956232	A	T	0.147	-0.020	0.005	1.66E-05	0.979	0.011	0.062
**rs11612312**	12	52349088	T	C	0.806	0.019	0.004	1.37E-06	0.958	0.010	1.41E-05
**rs11644362**	16	12994097	T	C	0.459	0.014	0.003	2.06E-05	0.989	0.008	0.163
**rs11668122**	19	57516240	A	G	0.920	0.028	0.007	3.38E-05	1.013	0.015	0.374
**rs11691770**	2	168633442	T	G	0.039	0.037	0.008	8.52E-06	0.992	0.020	0.680
**rs11751387**	6	124633499	T	C	0.097	0.021	0.005	3.07E-05	1.019	0.013	0.133
**rs12062152**	1	234267329	A	G	0.039	0.035	0.008	2.10E-05	0.992	0.019	0.662
**rs12143280**	1	211547522	T	C	0.944	0.038	0.008	6.46E-07	1.002	0.017	0.888
**rs12147610**	14	55190709	T	C	0.306	-0.019	0.005	4.67E-05	1.005	0.011	0.653
**rs12152057**	21	25056215	A	C	0.674	0.015	0.003	1.14E-05	1.001	0.009	0.940
**rs12298541**	12	66306441	A	C	0.356	0.016	0.003	2.28E-06	0.999	0.008	0.893
**rs12460988**	19	31162121	T	C	0.573	-0.014	0.003	1.67E-05	0.991	0.008	0.281
**rs12474324**	2	19346130	A	G	0.942	0.027	0.006	1.18E-05	1.002	0.015	0.902
**rs12995715**	2	63676678	C	G	0.239	-0.016	0.004	1.46E-05	1.007	0.009	0.451
**rs13235506**	7	53785701	T	G	0.955	-0.034	0.007	1.96E-06	1.038	0.020	0.059
**rs1328835**	13	109883183	C	G	0.944	0.027	0.007	3.20E-05	0.979	0.016	0.184

**rs13387164**	2	154144551	A	G	0.091	-0.023	0.005	5.98E-06	1.013	0.013	0.339
**rs1361360**	10	20795797	T	C	0.401	0.014	0.003	3.30E-05	0.996	0.008	0.654
**rs1462450**	18	49204707	A	G	0.892	0.020	0.005	1.11E-05	0.987	0.012	0.253
**rs1520724**	12	94865477	T	C	0.586	0.013	0.003	4.65E-05	0.995	0.008	0.496
**rs1556477**	9	20735284	A	G	0.634	0.014	0.003	3.42E-05	1.000	0.008	0.975
**rs16847494**	3	137532063	C	G	0.754	0.015	0.004	3.38E-05	0.989	0.009	0.215
**rs17005492**	4	140960772	T	C	0.785	0.018	0.004	2.01E-06	0.990	0.010	0.270
**rs17331012**	4	12144655	A	G	0.129	-0.023	0.005	1.10E-06	1.002	0.012	0.864
**rs17693963**	6	27710165	A	C	0.897	-0.026	0.005	9.25E-07	1.071	0.013	1.24E-07
**rs17766081**	4	68064345	C	G	0.558	0.013	0.003	4.79E-05	0.992	0.009	0.395
**rs2017279**	10	3534725	A	G	0.679	0.016	0.003	3.15E-06	0.985	0.009	0.080
**rs2058382**	7	69579024	A	G	0.590	-0.015	0.003	6.12E-06	1.007	0.008	0.365
**rs2075677**	20	47701024	A	G	0.774	0.021	0.004	1.88E-08	0.991	0.009	0.328
**rs210896**	6	11729440	T	G	0.765	0.017	0.004	1.01E-05	1.000	0.010	0.993
**rs2127737**	16	73639443	T	C	0.843	0.018	0.004	2.08E-05	0.991	0.011	0.374
**rs2212682**	21	21527469	T	G	0.847	-0.019	0.005	3.94E-05	0.997	0.012	0.797
**rs2321300**	2	134582082	A	G	0.146	-0.017	0.004	3.41E-05	0.995	0.010	0.645
**rs2409722**	8	11039816	T	G	0.463	0.013	0.003	2.06E-05	1.004	0.008	0.591
**rs258677**	7	81733428	T	G	0.375	0.017	0.003	9.46E-08	0.997	0.008	0.747
**rs2597455**	4	163307671	T	G	0.623	-0.014	0.003	1.78E-05	1.006	0.008	0.482
**rs281288**	15	47681703	T	G	0.345	-0.016	0.004	1.99E-05	1.041	0.010	3.26E-05
**rs2819873**	10	81906119	T	C	0.052	0.030	0.007	1.32E-05	0.996	0.017	0.805
**rs2911244**	16	89521860	A	G	0.478	0.013	0.003	4.29E-05	0.990	0.008	0.221
**rs3104708**	16	10071193	A	G	0.118	-0.021	0.005	8.58E-06	0.996	0.012	0.723
**rs35238**	5	52293721	A	G	0.039	-0.040	0.010	4.75E-05	1.013	0.024	0.577
**rs37241**	5	89853342	C	G	0.058	0.036	0.009	4.73E-05	0.974	0.018	0.146
**rs3756290**	5	130951750	A	G	0.237	-0.016	0.004	1.44E-05	1.008	0.010	0.467
**rs3828653**	5	114482595	C	G	0.970	0.035	0.009	4.76E-05	1.028	0.021	0.184

**rs4570163**	8	123451096	T	C	0.702	-0.014	0.003	1.54E-05	1.006	0.008	0.510
**rs4589952**	3	95702105	T	C	0.155	-0.021	0.004	1.55E-06	1.017	0.011	0.131
**rs4762896**	12	22401911	T	C	0.265	-0.016	0.004	3.51E-05	1.001	0.010	0.950
**rs4792196**	17	11820388	A	T	0.188	0.018	0.004	4.18E-05	0.992	0.011	0.481
**rs4810682**	20	46387986	A	G	0.821	-0.021	0.005	3.61E-05	0.994	0.011	0.598
**rs4842283**	12	80822975	A	C	0.903	-0.029	0.006	1.94E-07	1.001	0.015	0.955
**rs6073597**	20	43629512	T	C	0.552	0.014	0.003	7.96E-06	0.992	0.008	0.289
**rs6089930**	20	61359001	A	G	0.457	-0.014	0.003	1.32E-05	0.999	0.008	0.938
**rs6587766**	1	57708088	T	C	0.041	0.047	0.009	2.52E-07	0.983	0.024	0.481
**rs6598440**	15	101795040	A	G	0.968	0.032	0.008	2.68E-05	1.040	0.019	0.039
**rs6704609**	2	122950483	C	G	0.955	0.028	0.007	3.65E-05	0.969	0.018	0.083
**rs6772840**	3	43854320	A	T	0.090	0.025	0.006	5.83E-06	0.988	0.015	0.392
**rs6813656**	4	82380368	T	C	0.968	-0.045	0.010	7.74E-06	0.991	0.022	0.682
**rs7072297**	10	44462360	C	G	0.843	0.018	0.004	3.55E-05	0.988	0.011	0.268
**rs7149000**	14	41897318	A	G	0.459	-0.014	0.003	4.77E-06	1.026	0.008	0.002
**rs7239776**	18	52578599	A	T	0.530	-0.016	0.003	3.31E-07	1.016	0.008	0.056
**rs7445606**	5	152200109	A	C	0.381	-0.013	0.003	4.86E-05	1.013	0.008	0.114
**rs7487682**	12	48579665	T	G	0.410	-0.013	0.003	3.43E-05	1.008	0.008	0.354
**rs7584895**	2	29229016	T	C	0.519	-0.014	0.003	1.15E-05	1.009	0.008	0.258
**rs7939430**	11	134631823	A	G	0.149	-0.019	0.004	3.40E-05	1.012	0.011	0.278
**rs7952069**	11	12917033	T	C	0.890	0.021	0.005	5.66E-06	1.000	0.013	0.977
**rs8073904**	17	79095144	A	G	0.855	-0.021	0.005	1.24E-05	1.009	0.013	0.495
**rs8180800**	7	4161793	T	C	0.388	0.013	0.003	4.97E-05	0.991	0.008	0.331
**rs903834**	11	10814116	A	G	0.407	0.015	0.003	1.17E-05	0.987	0.008	0.115
**rs905456**	3	30746255	A	G	0.429	-0.014	0.003	1.48E-05	1.005	0.008	0.525
**rs9291932**	5	68043243	T	C	0.718	0.015	0.004	4.28E-05	1.002	0.009	0.866
**rs929814**	19	30340412	A	G	0.638	0.013	0.003	3.33E-05	1.006	0.008	0.465
**rs9528554**	13	63282834	T	C	0.784	0.016	0.004	9.59E-06	1.013	0.009	0.155

**rs976337**	11	95011645	T	C	0.597	0.014	0.003	5.27E-06	0.996	0.008	0.654
**rs9840016**	3	159899731	T	C	0.778	-0.017	0.004	1.68E-05	0.985	0.010	0.114
**rs9956636**	18	31304974	T	C	0.377	-0.013	0.003	4.59E-05	1.023	0.008	0.005

Note. SNPs for subjective wellbeing are from Okbay [1] and have been extracted from the most recent GWAS of Major Depressive Disorder (MDD) [12]. None is associated with MDD in the current GWAS at the genome-wide level of significance. A1 = effect allele, A2 = non-effect allele, EAF = effect allele frequency. rG between subjective wellbeing and MDD was -0.65 (SE = 0.04) [12].

Table S5. Power calculation for two-sample Mendelian randomisation

Exposure	Variance Explained by the Instrument (r^2)	Causal effect we have 80% power to detect
**Wellbeing**	0.01%	0.971 to 0.481
	0.05%	0.435 to 0.215
	0.5%	0.138 to 0.068
	1%	0.097 to 0.049
**BMI**	2.7% [2]	0.031
**Waist-to-Hip ratio**	1.4% [3]	0.044
**Body Fat**	0.58% [4]	0.068
**HDL Cholesterol**	1.6% [5]	0.041
**LDL Cholesterol**	2.4% [5]	0.033
**Total Cholesterol**	2.6% [5]	0.032
**Waist circumference, diastolic blood pressure, systolic blood pressure, CAD, MI**	0.05%	0.230
	0.5%	0.073
	1%	0.052
	2%	0.037

Note. Power calculations were conducted using Burgess' online calculator [1]. Causal effect refers to the change in outcome in SD units per SD change in exposure. Where possible, variance explained from the original GWAS paper was used. For waist circumference, blood pressure, CAD and MI r^2 was not reported so we give a range of values. For subjective wellbeing, variance explained at $p<5x10^{-5}$ was unknown, therefore a range of values are given. For all calculations we used the outcome sample size. For subjective wellbeing we present the range of values we can detect given the smallest sample size (83198 for LDL cholesterol) and the largest (339224 for BMI). All others had subjective wellbeing as the outcome so sample size is 298420.

Table S6. SNPs associated with BMI at the genome-wide level of significance

SNP Name	Effect Allele	Non-effect Allele	MAF	Beta	SE	p-value
**rs1000940**	G	A	0.225	0.018	0.003	1.81E-08
**rs10132280**	A	C	0.333	-0.022	0.003	1.40E-11
**rs1016287**	T	C	0.325	0.023	0.003	4.36E-12
**rs10182181**	A	G	0.500	-0.031	0.003	8.07E-26
**rs10733682**	A	G	0.425	0.019	0.003	2.46E-10
**rs10938397**	A	G	0.567	-0.040	0.003	1.42E-40
**rs10968576**	G	A	0.292	0.025	0.003	2.34E-14
**rs11030104**	A	G	0.800	0.042	0.004	6.66E-30
**rs11057405**	A	G	0.092	-0.030	0.005	1.22E-08
**rs11126666**	G	A	0.692	-0.020	0.003	1.32E-09
**rs11165643**	C	T	0.425	-0.022	0.003	1.43E-13
**rs11191560**	T	C	0.942	-0.031	0.005	2.08E-09
**rs11583200**	C	T	0.375	0.017	0.003	6.00E-09
**rs1167827**	A	G	0.458	-0.020	0.003	1.98E-10
**rs11688816**	A	G	0.542	-0.015	0.003	3.80E-07
**rs11727676**	C	T	0.075	-0.037	0.006	6.25E-09
**rs11847697**	T	C	0.042	0.037	0.007	2.61E-07
**rs9581854**	C	T	0.767	-0.030	0.005	9.28E-11
**rs12286929**	G	A	0.433	0.021	0.003	5.44E-13
**rs12401738**	A	G	0.425	0.020	0.003	2.04E-10
**rs12429545**	G	A	0.900	-0.032	0.004	3.15E-13
**rs12446632**	A	G	0.133	-0.040	0.004	1.81E-19
**rs12566985**	G	A	0.425	0.024	0.003	1.95E-15
**rs12885454**	C	A	0.633	0.020	0.003	9.09E-11
**rs12940622**	A	G	0.458	-0.018	0.003	3.64E-10
**rs13021737**	A	G	0.125	-0.060	0.004	5.44E-54
**rs13078960**	T	G	0.817	-0.029	0.004	1.42E-14
**rs13107325**	C	T	0.883	-0.047	0.007	1.06E-12
**rs13191362**	A	G	0.800	0.029	0.005	1.09E-09
**rs13201877**	A	G	0.917	-0.024	0.004	4.29E-08
**rs1441264**	A	G	0.550	0.017	0.003	2.96E-08
**rs1460676**	T	C	0.783	-0.021	0.004	4.98E-08
**rs1516725**	T	C	0.092	-0.045	0.004	1.39E-24
**rs1528435**	T	C	0.583	0.018	0.003	4.77E-09
**rs1558902**	A	T	0.450	0.081	0.003	1.13E-156
**rs16851483**	G	T	0.908	-0.048	0.008	1.85E-10
**rs16907751**	C	T	0.958	0.033	0.006	2.13E-07
**rs16951275**	C	T	0.225	-0.030	0.004	2.04E-18
**rs17001654**	C	G	0.842	-0.030	0.005	5.03E-09
**rs17024393**	C	T	0.042	0.061	0.008	1.64E-13
**rs17094222**	C	T	0.208	0.025	0.004	2.19E-11
**rs17203016**	G	A	0.200	0.021	0.004	3.41E-08
**rs17405819**	C	T	0.367	-0.022	0.003	1.17E-11
**rs17724992**	A	G	0.692	0.020	0.003	7.79E-09
**rs1808579**	T	C	0.475	-0.016	0.003	4.25E-08

**rs1928295**	C	T	0.425	-0.018	0.003	4.32E-10
**rs2033529**	G	A	0.289	0.018	0.003	1.39E-08
**rs2033732**	C	T	0.758	0.018	0.003	2.26E-07
**rs205262**	A	G	0.733	-0.021	0.003	2.70E-10
**rs2075650**	A	G	0.858	0.026	0.004	3.21E-09
**rs2080454**	A	C	0.608	-0.017	0.003	8.60E-09
**rs2112347**	G	T	0.375	-0.025	0.003	1.96E-17
**rs2121279**	T	C	0.117	0.024	0.004	1.92E-08
**rs2176040**	G	A	0.392	-0.015	0.003	1.33E-06
**rs2176598**	T	C	0.608	-0.015	0.003	3.47E-08
**rs2207139**	G	A	0.200	0.019	0.003	8.06E-31
**rs2245368**	T	C	0.100	0.045	0.004	7.01E-08
**rs2287019**	C	T	0.758	-0.029	0.005	1.68E-18
**rs2365389**	C	T	0.850	0.035	0.004	1.35E-10
**rs2650492**	A	G	0.658	0.020	0.003	1.29E-09
**rs2820292**	A	C	0.308	0.021	0.003	5.45E-10
**rs2836754**	C	T	0.492	-0.018	0.003	1.61E-08
**rs29941**	A	G	0.650	0.017	0.003	2.20E-08
**rs3101336**	T	C	0.333	-0.018	0.003	6.49E-26
**rs3736485**	A	G	0.351	-0.032	0.003	4.52E-08
**rs3810291**	A	G	0.425	0.016	0.003	6.35E-16
**rs3817334**	C	T	0.625	0.029	0.004	1.17E-17
**rs3849570**	A	C	0.550	-0.026	0.003	1.93E-08
**rs3888190**	A	C	0.367	0.018	0.003	3.45E-25
**rs4256980**	G	C	0.358	0.031	0.003	8.35E-12
**rs4740619**	T	C	0.725	0.021	0.003	6.36E-09
**rs4787491**	A	G	0.533	0.017	0.003	2.34E-06
**rs492400**	T	C	0.386	-0.015	0.003	4.87E-07
**rs543874**	G	A	0.675	-0.015	0.003	2.29E-40
**rs6091540**	C	T	0.267	0.050	0.004	2.14E-08
**rs6465468**	G	T	0.725	0.019	0.003	2.44E-06
**rs6477694**	C	T	0.675	-0.016	0.003	1.71E-08
**rs6567160**	C	T	0.358	0.017	0.003	6.68E-59
**rs657452**	A	G	0.283	0.056	0.004	2.12E-13
**rs6804842**	A	G	0.417	0.023	0.003	8.02E-10
**rs7138803**	G	A	0.425	-0.018	0.003	5.12E-26
**rs7141420**	T	C	0.558	-0.032	0.003	8.66E-15
**rs7164727**	T	C	0.617	0.023	0.003	3.92E-09
**rs7239883**	G	A	0.775	0.019	0.003	3.14E-07
**rs7243357**	G	T	0.317	0.015	0.003	9.14E-09
**rs758747**	C	T	0.133	-0.022	0.004	1.51E-10
**rs7599312**	G	A	0.733	-0.023	0.004	4.73E-11
**rs7715256**	G	T	0.708	0.021	0.003	8.85E-09
**rs7899106**	A	G	0.450	0.017	0.003	1.27E-08
**rs7903146**	T	C	0.950	-0.038	0.007	1.10E-12
**rs9374842**	T	C	0.250	-0.024	0.003	7.20E-09
**rs9400239**	C	T	0.742	0.020	0.003	6.77E-08
**rs9540493**	G	A	0.700	0.017	0.003	3.95E-09
**rs9641123**	G	C	0.550	-0.018	0.003	1.83E-07

**rs977747**	T	G	0.608	-0.019	0.004	2.18E-08
**rs9914578**	G	C	0.467	0.017	0.003	2.07E-08
**rs9925964**	G	A	0.167	0.020	0.004	9.35E-11

List of SNPs associated with BMI [1] used for one-sample Mendelian Randomisation.

Table S7. Regression dilution I^2 statistic for the heterogeneity of SNP-exposure effects.

Exposure	I^2
**Subjective wellbeing**	0.367
**BMI**	0.913
**Body Fat**	0.565
**Waist-to-Hip Ratio**	0.575
**Waist Circumference**	0.882
**CAD**	0.905
**MI**	0.903
**LDL Cholesterol**	0.986
**HDL Cholesterol**	0.971
**Total Cholesterol**	0.978
**Diastolic Blood Pressure**	0.774
**Systolic Blood Pressure**	0.689

Note: I^2 is a measure of the regression dilution of all SNP-exposure effects.

Regression dilution bias occurs when the SNPs are weakly associated with the exposure. This is also known as the `NO Measurement Error' (NOME) assumption. The I^2 statistic for the SNP-exposure (GX) effects is a measure of NOME violation. In order to conduct MR Egger regression I^2 should be greater than 0.9 or else simulation extrapolation (SIMEX) correction should be applied [13].

Table S8. Tests of heterogeneity for physical health traits as the exposure and subjective wellbeing as the outcome.

Exposure	Method	Q	df	P-value
**BMI**	MR Egger	33.96	23	0.066
	Inverse-Variance Weighted	34.24	24	0.081
**Waist-to-hip Ratio**	MR Egger	35.12	34	0.415
	Inverse-Variance Weighted	36.03	35	0.420
**Waist circumference**	MR Egger	85.08	38	<0.001
	Inverse-Variance Weighted	90.64	39	<0.001
**Body Fat**	MR Egger	11.88	7	0.105
	Inverse-Variance Weighted	13.05	8	0.110
**HDL cholesterol**	MR Egger	76.9	65	0.148
	Inverse-Variance Weighted	78.4	65	0.141
**LDL cholesterol**	MR Egger	111.3	82	0.017
	Inverse-Variance Weighted	110.1	83	0.025
**Total cholesterol**	MR Egger	102.3	76	0.024
	Inverse-Variance Weighted	101	77	0.034
**CAD**	MR Egger	145	76	0.000
	Inverse-Variance Weighted	149.4	77	0.000
**Myocardial infarction**	MR Egger	29.95	13	0.005
	Inverse-Variance Weighted	27.81	14	0.015
**Diastolic blood pressure**	MR Egger	59.52	41	0.031
	Inverse-Variance Weighted	61.04	42	0.029
**Systolic blood pressure**	MR Egger	47.08	34	0.067
	Inverse-Variance Weighted	47.88	35	0.072

Note: df = degrees of freedom where degrees of freedom is equal to the number of SNPs -1. Q = Cochran's Q, a test of heterogeneity or dispersion in the SNP effects. Separate clusters of SNP effects suggest that they are acting through different pathways. This is an indicator that there might be a pleiotropic pathway.

Table S9. Tests of directional pleiotropic effects of the genetic instruments for physical health on subjective wellbeing.

Exposure	Intercept (95% CI)	P-value	MR-PRESSO Global Test	P-value
**BMI**	0.002 (-0.0001, 0.005)	0.070	157.36	<0.001
**Waist-to-hip ratio**	-0.003 (-0.008, 0.003)	0.353	38.74	0.387
**Waist circumference**	0.005 (-0.001, 0.010)	0.123	95.59	<0.001
**Body fat**	0.008 (-0.012, 0.028)	0.434	16.70	0.105
**HDL cholesterol**	-0.0003 (-0.002, 0.001)	0.769	115.17	0.019
**LDL cholesterol**	-0.001 (-0.003, 0.0003)	0.127	82.80	0.155
**Total cholesterol**	-0.0004 (-0.002, 0.002)	0.690	105.93	0.035
**CAD**	0.001 (-0.002, 0.003)	0.685	49.17	0.033
**Myocardial infarction**	-0.0001 (-0.006, 0.006)	0.994	42.49	0.002
**Diastolic blood pressure**	-0.002 (-0.006, 0.002)	0.312	63.93	0.029
**Systolic blood pressure**	-0.002 (-0.007, 0.003)	0.452	50.38	0.074

Note: Reported intercepts are the MR Egger intercept.

The MR Egger intercept showed no evidence for directional pleiotropy. Several phenotypes showed a significant MR-PRESSO global test. In this situation, outlier tests were run to correct for pleiotropic outliers. If distortion tests identified significantly different estimates following adjustment for outliers, then MR-PRESSO outlier corrected causal estimates are presented in Figure 3. Only the exposures BMI, waist circumference and HDL cholesterol had significant global tests and outlier tests.

Table S10. Linear regressions between BMI and subjective wellbeing in the UK Biobank sample

	Mean (SD)	Beta (95% CI)	N	p-value
**Happiness**	4.45 (0.70)	-0.001 (-0.002, -0.001)	110,347	0.002
**Satisfaction with work**	4.40 (0.87)	-0.001 (-0.002, 0.000)	75,519	0.097
**Satisfaction with health**	4.25 (0.87)	-0.048 (-0.047, -0.049)	110,388	<0.001
**Satisfaction with finances**	4.31 (0.94)	-0.021 (-0.020, -0.022)	110,247	<0.001
**Satisfaction with friends**	4.76 (0.74)	0.002 (0.001, 0.003)	109,550	0.001
**Satisfaction with family**	4.79 (0.90)	-0.000 (-0.001, 0.001)	109,712	0.938

Note. Means and standard deviations for the subjective wellbeing measures (scored from 1-6 with 6 being high wellbeing). Mean subjective wellbeing values show some negative skew but none have skew less than -1. Linear regressions were conducted to test the observational association between BMI and subjective wellbeing in our UK Biobank sample controlling for age, sex and SEP. BMI was negatively associated with all measures of subjective wellbeing apart from job satisfaction and satisfaction with family where there was no clear association and satisfaction with friends where the association was positive.

Table S11. Results of one-sample MR of BMI (exposure) on subjective wellbeing (outcome) in the independent UK Biobank sample controlling for age, sex and 10 principal components.

Outcome	Beta (95% CI)	P-value
**Happiness**	0.001 (-0.007, 0.009)	0.810
**Satisfaction with work**	0.005 (-0.007, 0.016)	0.417
**Satisfaction with health**	-0.037 (-0.046, -0.027)	<0.001
**Satisfaction with finances**	-0.003 (-0.014, 0.007)	0.516
**Satisfaction with friends**	0.003 (-0.005, 0.011)	0.515
**Satisfaction with family**	0.002 (-0.008, 0.012)	0.722

References

1 Okbay A, Baselmans BM, De Neve J-E, *et al.* Genetic variants associated with subjective well-being, depressive symptoms, and neuroticism identified through genome-wide analyses. *Nat Genet* 2016;**48**:624–33.

2 Locke AE, Kahali B, Berndt SI, *et al.* Genetic studies of body mass index yield new insights for obesity biology. *Nature* 2015;**518**:197–206.

3 Hemani G, Zheng J, Wade KH, *et al.* MR-Base: a platform for systematic causal inference across the phenome using billions of genetic associations. *bioRxiv* 2016;:078972.

4 Frayling TM, Timpson NJ, Weedon MN, *et al.* A common variant in the FTO gene is associated with body mass index and predisposes to childhood and adult obesity. *Science* 2007;**316**:889–894.

5 Loos RJF, Yeo GSH. The bigger picture of FTO—the first GWAS-identified obesity gene. *Nat Rev Endocrinol* 2014;**10**:51–61. doi:10.1038/nrendo.2013.227

6 CARDIoGRAMplusC4D Consortium, others. A comprehensive 1000 Genomes-based genome-wide association meta-analysis of coronary artery disease. *Nat Genet* 2015;**47**:1121–49.

7 Global Lipids Genetics Consortium, others. Discovery and refinement of loci associated with lipid levels. *Nat Genet* 2013;**45**:1274–1283.

8 Wain LV, Vaez A, Jansen R, *et al.* Novel blood pressure locus and gene discovery using genome-wide association study and expression data sets from blood and the kidney. *Hypertension* 2017;:HYPERTENSIONAHA–117.

9 Shungin D, Winkler TW, Croteau-Chonka DC, *et al.* New genetic loci link adipose and insulin biology to body fat distribution. *Nature* 2015;**518**:187–96. doi:10.1038/nature14132

10 Lu Y, Day FR, Gustafsson S, *et al.* New loci for body fat percentage reveal link between adiposity and cardiometabolic disease risk. *Nat Commun* 2016;**7**:10495.

11 Pierce BL, Ahsan H, VanderWeele TJ. Power and instrument strength requirements for Mendelian randomization studies using multiple genetic variants. *Int J Epidemiol* 2011;**40**:740–52. doi:10.1093/ije/dyq151

12 Wray NR, Sullivan PF, others. Genome-wide association analyses identify 44 risk variants and refine the genetic architecture of major depression. *Nat Genet* 2018;**50**:668–81.

13 Bowden J, Del Greco M F, Minelli C, *et al.* Assessing the suitability of summary data for two-sample Mendelian randomization analyses using MR-Egger regression: the role of the I 2 statistic. *Int J Epidemiol* 2016;**45**:1961–1974.
